# Stabilising spatiotemporal dynamics of mussel–algae coupled map lattices model via proportional–differential control

**DOI:** 10.1007/s00285-025-02292-z

**Published:** 2025-10-06

**Authors:** Yanhua Zhu, Xiangyi Ma, Jinliang Wang, Federico Frascoli, Tonghua Zhang

**Affiliations:** 1 Department of Mathematics, Beihang University, Changping, Beijing 100191 People’s Republic of China; 2 Department of Mathematics, Swinburne University of Technology, Hawthorn, Melbourne, VIC 3122 Australia

**Keywords:** Mussel–algae system, Coupled map lattices model, Turing instability, Proportional–differential control, 92C15, 92B05, 92D25

## Abstract

The mussel–algae (M–A) system plays a crucial role in maintaining the balance of marine aquaculture ecosystems. Mussels filter algae from the water as a food source, while algae produce oxygen through photosynthesis and contribute to nutrient cycling. Fluctuations in the density and spatial distribution of algae populations can significantly impact the growth and reproduction of mussels, and conversely, mussels can influence algae dynamics, thereby potentially altering the equilibrium of the system. This study adopts a practical perspective, simultaneously considering the effects of self-diffusion and cross-diffusion, and establishes a spatiotemporally discretised coupled map lattices (CMLs) model for the M–A system. Utilising linear stability analysis, bifurcation theory, and the centre manifold theorem, we explore the stability and classification of fixed points within the CMLs model, as well as the parameter conditions that give rise to flip and Turing bifurcations. Numerical simulations demonstrate the rich temporal dynamics and spatiotemporal patterns induced by five different mechanisms. Notably, we introduce a proportional–differential (PD) control into the CMLs model for the first time. Through numerical simulations, we validate that the PD control can delay the occurrence of the flip bifurcation, thereby preventing multi-period oscillations and chaos in algal population density, which could lead to system instability. Moreover, the PD control can reduce the Turing instability region and adjust the Turing pattern types induced by the five mechanisms, thus ensuring a uniform spatiotemporal distribution of the algal population and contributing to the stability of the ecosystem.

## Introduction

Mussels are among the most abundant species in coastal zones (Koppel et al. 2005; Williams et al. 2024). In addition to serving as a crucial resource within the food web (Martins et al. 2024; Liénart et al. 2025), they significantly contribute to the stability of marine aquaculture ecosystems. As filter-feeding organisms, mussels obtain nutrients by filtering water through their bodies. Algae, a primary food source for mussels, facilitate oxygen production and nutrient cycling through photosynthesis. The interaction between mussels and algae plays a critical role in ecosystem stability, water purification, and marine aquaculture (Newell 2004; Stadmark and Conley 2011). This interaction can be mathematically characterised using reaction–diffusion equations (Song et al. 2017; Cangelosi et al. 2015), which have also been extensively employed to describe a diverse range of biological and ecological systems, including predator–prey dynamics (Song et al. 2024; Peng et al. 2024; Song et al. 2021), aquatic ecosystems (Zhao et al. 2022), and pattern formation (Yang et al. 2020; Yan et al. 2020; Jia et al. 2019). In Turing (1952), Turing introduced the effect of self-diffusion on pattern formation in reaction–diffusion systems in his seminal paper, proposing the concept of Turing instability. This phenomenon refers to a situation in which an initially stable homogeneous solution of a reaction–diffusion system becomes unstable due to the presence of self-diffusion terms. Over the past few decades, Turing instability has found applications across various fields, including biology (Han et al. 2023; Wang et al. 2023; Zhang and Zang 2014), physics (Karachalios et al. 2024), chemistry (Zhao et al. 2024; Fu et al. 2021), and embryogenesis (Wang et al. 2015; Zhao et al. 2021).

Research on the mussel–algae (M–A) reaction–diffusion model has yielded numerous findings (Shen and Wei 2019; Song et al. 2017; Shen and Wei 2022; Zhong et al. 2021). For instance, in Ref. Song et al. (2017), Song et al. investigated pattern formation in a reaction–diffusion M–A model, focusing on the Turing–Hopf bifurcation and its role in enhancing spatial pattern diversity through theoretical analysis and numerical simulations. In Ref. Shen and Wei (2019), the authors analyzed the spatiotemporal patterns and bifurcations in a delayed reaction–diffusion M–A system, revealing how Turing–Hopf interactions diversify population distributions. Notably, the aforementioned studies on the M–A reaction–diffusion model have primarily considered the effects of self-diffusion. In reality, reaction–diffusion models encompass not only self-diffusion but also cross-diffusion (Yang et al. 2020; Tang et al. 2016), as well as super-diffusion, sub-diffusion, and other mechanisms, depending on the spatial movement of system variables. When mussel density becomes excessively high, they can deplete significant quantities of algae, leading to local resource scarcity that drives algae to diffuse towards areas of lower density through competition or other mechanisms. These interactions cannot be adequately captured by self-diffusion alone; therefore, it is essential to account for cross-diffusion.

On the other hand, the aforementioned studies primarily focus on continuous reaction–diffusion systems. In reality, the collection of growth data for mussels and algae occurs in a temporally discontinuous manner, while the spatial distribution of these organisms is inherently discrete. Moreover, the simulation algorithms for spatial patterns often rely on discrete forms of their respective continuous systems. Given this analysis, it is more aligned with real-world conditions to consider a spatiotemporally discrete M-A reaction–diffusion model, specifically a coupled map lattice (CMLs) model. This approach more accurately reflects the discrete nature of empirical data collection and the spatial distribution of biological entities. A substantial body of research has been accumulated on CMLs model, as detailed in references (Huang and Zhang 2016; Huang et al. 2017; Salman and Han 2023; Wang et al. 2019; Zhu et al. 2025). According to these studies, CMLs models display richer dynamical behaviors compared to their continuous counterparts. Research on CMLs models can be broadly categorised into two main aspects. On one hand, studies on spatially homogeneous systems concentrate on stability analysis, flip bifurcation, and Neimark-Sacker bifurcation. Among these, flip bifurcations are often regarded as transitions from period-doubling behavior to chaotic states. As the system’s nonlinearity increases (for instance, due to competition between mussel and algae populations or environmental stochasticity), period-doubling leads to unstable fluctuations in algae population density, further driving the system towards chaos. The onset of chaos results in unpredictability in algae population density. On the other hand, research on spatially heterogeneous systems primarily centres on Turing bifurcations, which can induce population instability and trigger the formation of spatiotemporal patterns, leading to uneven spatial and temporal distributions of algae. In marine aquaculture ecosystems, unstable fluctuations and uneven distributions of algae population density can severely impact the growth and reproduction of mussels, thereby threatening the stability of the ecosystem. Through this analysis, a critical and challenging question arises: can certain control strategies be applied to delay the occurrence of flip and Turing bifurcations, thereby stabilising algae population density and ensuring a uniform spatial distribution? Addressing this question could provide valuable insights into maintaining ecological balance and enhancing the sustainability of marine aquaculture systems.

To this extent and in recent decades, the development of control strategies has progressed rapidly, finding extensive applications in numerous critical fields such as ecology, infectious diseases, economics, complex networks, and traffic (Abbasi 2024; Chang et al. 2022; Huang et al. 2018; Gao and Zhang 2022; Xu et al. 2024; Wu et al. 2023; Wang et al. 2024). Currently, various control strategies, including hybrid control, proportional–differential control, and delayed feedback control, have been incorporated into ordinary differential equation models and reaction–diffusion models to regulate their dynamical behaviors. For instance, in Ref. Cui et al. (2024), Cui et al. examined the regulation of Hopf bifurcation in a delayed 5D BAM neural network through two hybrid control strategies, demonstrating how parameter tuning can affect both the stability domain and the onset of bifurcation. Similarly, in reference (Tao et al. 2022), Tao et al. proposed a distributed time-delay feedback control strategy that maintains equilibrium properties, ensures continuous stability intervals, and effectively enhances the bifurcation threshold. For further related studies, please refer to Ju et al. (2024); Ding et al. (2016); Luan et al. (2024). Notably, PD control with dual-parameter adjustment have received significant attention due to their operational flexibility. For example, in reference (He et al. 2024), the authors investigated the spatiotemporal dynamics of the Gierer-Meinhardt model using Hopf bifurcation and Turing instability theorems, proposing control strategies to delay Hopf bifurcation and widen the thresholds of Turing instability. Additionally, in reference (Li et al. 2023; Lu et al. 2023), a fractional-order PD control was developed to regulate spatiotemporal dynamics.

Inspired by the analysis presented above, this paper innovatively introduces a PD control into the CMLs model to regulate the occurrence of flip bifurcation, Turing bifurcation, and the adjustment of pattern types. Through this approach, we aim to stabilise algal population density, ensure a uniform spatiotemporal distribution of algae, and ultimately maintain the stability of marine aquaculture ecosystems. This method bridges the gap between control theory and ecological modelling, offering a potential solution to mitigate the adverse effects of population fluctuations and spatial heterogeneity in marine ecosystems. Based on this comprehensive analysis, the primary contributions of this paper are outlined as follows:

- From an application standpoint, we concurrently examine the impacts of self-diffusion and cross-diffusion to formulate a CMLs model tailored for the M-A system. A PD control is integrated into the CMLs model to modulate its dynamic characteristics.
- Employing methods such as linear stability analysis, bifurcation theory, and the center manifold theorem, we investigate the stability and nature of fixed points within the controlled CMLs model. We identify the parameter conditions leading to flip bifurcation and Turing bifurcation in the system.
- By performing numerical simulations, we illustrate the diverse temporal dynamics of the CMLs model and exhibit spatiotemporal patterns prompted by five Turing instability mechanisms, including pure-self-diffusion-Turing instability, pure-cross-diffusion-Turing instability, flip-self-diffusion-Turing instability, flip-cross-diffusion-Turing instability, and chaos-self-diffusion-Turing instability. Additionally, we confirm that implementing the PD control can postpone the onset of flip bifurcation and minimize the Turing instability region. This enhances the stability of algae population density, ensuring an even spatiotemporal distribution of algae, which supports mussel growth, reproduction, and the stability of marine aquaculture ecosystems.
- The variations in flip and Turing bifurcation thresholds in response to PD control parameters are described. Meanwhile, the evolution of the Turing patterns under different control parameters is discussed. This facilitates the selection of optimal control settings to achieve specific dynamic outcomes.

This paper is structured as follows. Section 2 presents the model description and outlines the methodology for constructing the CMLs model. Section 3 analyzes the stability of the homogeneous stationary state. The bifurcations of the homogeneous stationary state are explored in Sect. 4. Numerical simulations that validate the theoretical results are discussed in Sect. 5. In conclusion, Sect. 6 provides a concise summary of the paper’s main points.

## Model description and establishment of the CMLs model

**Fig. 1:**
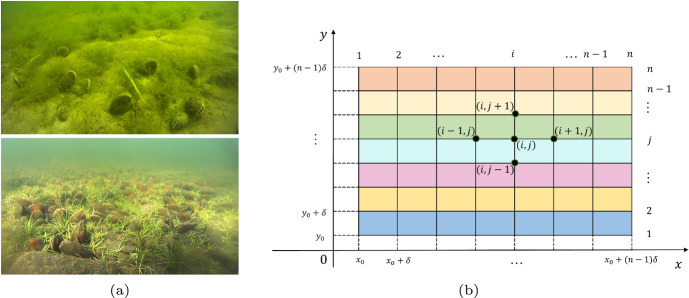
**a** Images of adult mussels in the experimental site (Image source: Ref. Harrison et al. (2024)); **b** the lattice framework consisting of $n \times n$ lattice sites

The mussel image (Fig. 1a) depicts the interactions between mussels and algae. In this section, we develop a CMLs model for the M-A system. Within a two-dimensional rectangular domain, we define a lattice framework consisting of $n \times n$ lattice sites, as shown in Fig. 1b. In this study, each lattice site, denoted by (*i*, *j*), where $(i,j\in \{1,2, \cdots , n\})$, is assigned two variables: the mussel biomass $m_{(i,j,t)}$ and the algae biomass $a_{(i,j,t)}$ at time $t\in Z^{+}$. Across different locations, we assume the existence of localized reactions and spatial dispersions (Punithan et al. 2012; Mistro et al. 2012), with the mussel and algae biomass at each site evolving according to the temporal dynamics of the system.

From time *t* to $t+1$, the dynamic behaviors of mussels and algae are characterised by two distinct stages: a “reaction” stage followed by a “diffusion” stage (Huang and Zhang 2016; Punithan et al. 2012; Mistro et al. 2012; Díaz Rodrigues et al. 2012). The diffusion behavior occurs prior to the reaction process. By introducing time step $\tau$ to monitor changes in population density and spatial step $\delta$ for mussels and algae, we establish the equations governing the dispersal process:

1a $$\begin{aligned} M^{\prime }_{(i, j, t)}=M_{(i, j, t)}+\frac{\tau }{\delta ^2} D_{M} \nabla _d^2 M_{(i, j, t)}+\frac{\tau }{\delta ^2} D_{\bar{M}} \nabla _d^2 A_{(i, j, t)}, \end{aligned}$$

1b $$\begin{aligned} A^{\prime }_{(i, j, t)}=A_{(i, j, t)}+\frac{\tau }{\delta ^2} D_{\bar{A}} \nabla _d^2 M_{(i, j, t)}+\frac{\tau }{\delta ^2} D_{A} \nabla _d^2 A_{(i, j, t)}. \end{aligned}$$

where $D_M$ and $D_A$ are the self-diffusion coefficients, and $D_{\bar{M}}$ and $D_{\bar{A}}$ are the cross-diffusion coefficients, all of which are positive constants. Additionally, $M_{(i,j,t)}^{\prime }$ and $A_{(i,j,t)}^{\prime }$ denote the biomass of mussels and algae that will participate in the subsequent reaction stage. The diffusion is described by the discrete Laplacian operator $\nabla _d^2$ that is defined by

2 $$\begin{aligned} {\left\{ \begin{array}{ll}\nabla _d^2M_{(i,j,t)}=M_{(i+1,j,t)}+M_{(i-1,j,t)}+M_{(i,j+1,t)}+M_{(i,j-1,t)}-4M_{(i,j,t)},\\ \nabla _d^2A_{(i,j,t)}=A_{(i+1,j,t)}+A_{(i-1,j,t)}+A_{(i,j+1,t)}+A_{(i,j-1,t)}-4A_{(i,j,t)}.& \end{array}\right. } \end{aligned}$$

In the following, we establish the equations governing the reaction process. Firstly, starting from the dynamics equation for mussels, we formulate the model. As mussels grow by consuming algae, we employ $ceA'_{(i,j,t)}M'_{(i,j,t)}$ to describe the growth process of mussels, where *c* denotes the consumption rate constant and *e* represents the conversion efficiency. To reflect the mortality characteristics under limited resources, we use a Holling type II saturated mortality rate $d_M\frac{k_M}{k_M+M'_{(i,j,t)}}M'_{(i,j,t)}$, where $d_M$ is the maximum mortality rate and $k_M$ is the saturation density. Consequently, after a monitoring period of $\tau$, the dynamics equation for mussels can be characterised as follows:

3 $$\begin{aligned} M_{(i,j,t+1)}=M'_{(i,j,t)}+\tau \left[ c e A'_{(i,j,t)}M'_{(i,j,t)}-d_M\frac{k_M}{k_M+M'_{(i,j,t)}}M'_{(i,j,t)}\right] , \end{aligned}$$

Secondly, we formulate the dynamic equation characterising the concentration of algae. Generally, a water body can be divided into upper and lower layers of water, and this stratification can result from differences in temperature (thermal stratification), differences in salinity (saline stratification) or other environmental factors. We assume that the concentration of algae differs between the upper and lower layers and can be transferred through an exchange process. Let $A_{UP}$ denote the concentration of algae in the upper water layer, and let $\rho$ represent the exchange rate of algae concentration between the upper and lower water layers. Then, the exchange process of algae between the two layers can be described by $(A_{UP}-A')\rho$. Assuming the height of the lower water layer is *H*, the grazing consumption at point (*i*, *j*) and time *t* of algae by mussels can be defined as $\frac{c}{H}A'_{(i,j,t)}M'_{(i,j,t)}$. Moreover, the influence of water flow on the distribution of algae needs to be taken into account, which we represent by $V\left( \frac{\partial A'}{\partial i}+\frac{\partial A'}{\partial j}\right)$, where *V* denotes the advection speed. Thus, the dynamic equation for algae concentration can be described as follows:

4 $$\begin{aligned} \begin{aligned} A_{(i,j,t+1)}&=A'_{(i,j,t)}+\tau \Bigg [\left( A_{UP}-A'_{(i,j,t)}\right) \rho -\frac{c}{H}A'_{(i,j,t)}M'_{(i,j,t)}\\&\quad -V\left( \frac{\partial A'_{(i,j,t)}}{\partial i}+\frac{\partial A'_{(i,j,t)}}{\partial j}\right) \Bigg ]. \end{aligned} \end{aligned}$$

For equations (1)–(4), we can perform the following nondimensionalisation:

$$ m=\frac{M}{k_M},a=\frac{A}{A_{UP}},\tau =\frac{\tilde{\tau }}{d_M} $$

and define $\omega =\frac{ck_M}{H},$ $r=\frac{ceA_{UP}}{d_M}$, $\alpha =\frac{\rho }{\omega }$, $\gamma =\frac{d_M}{\omega },$ $d_{m_1}=\frac{D_M}{d_M}$, $d_{m_2}=\frac{D_{\bar{M}}A_{UP}}{d_M k_M},$ $\nu =\frac{V}{d_M},$ $d_{a_1}=\frac{D_{\bar{A}}k_{M}}{A_{UP}d_M},$ $d_{a_2}=\frac{D_A}{d_M}$. Additionally, we drop the tilde and use $\tau$ instead of $\tilde{\tau }$ for convenience. While algae are typically considered to be influenced by advection from tidal flow at larger spatial scales, they disperse as Brownian particles in fluid at smaller scales. Laboratory experiments have demonstrated that mussels exhibit active movement both within and between clusters (van de Koppel et al. 2008; Liu et al. 2013), suggesting that tidal flow advection has a negligible impact on mussel beds at small spatial scales. Therefore, it is reasonable to set $V=0$, and, from the preceding analysis, equations (1)–(4) can now be written in the form:

5 $$\begin{aligned} {\left\{ \begin{array}{ll} \begin{aligned} m^{\prime }(i, j, t)& =m(i, j, t)+\frac{\tau }{\delta ^2} d_{m_1} \nabla _d^2 m(i, j, t)+\frac{\tau }{\delta ^2} d_{m_2} \nabla _d^2 a(i, j, t),\\ a^{\prime }(i, j, t)& =a(i, j, t)+\frac{\tau }{\delta ^2} d_{a_1} \nabla _d^2 m(i, j, t)+\frac{\tau }{\delta ^2} d_{a_2} \nabla _d^2 a(i, j, t),\\ \nabla _d^2m_{(i,j,t)}& =m_{(i+1,j,t)}+m_{(i-1,j,t)}+m_{(i,j+1,t)}+m_{(i,j-1,t)}-4m_{(i,j,t)},\\ \nabla _d^2a_{(i,j,t)}& =a_{(i+1,j,t)}+a_{(i-1,j,t)}+a_{(i,j+1,t)}+a_{(i,j-1,t)}-4a_{(i,j,t)},\\ m_{(i,j,t+1)}& =m'_{(i,j,t)}+\tau \left[ r a'_{(i,j,t)}m'_{(i,j,t)}-\frac{m'_{(i,j,t)}}{1+m'_{(i,j,t)}}\right] ,\\ a_{(i,j,t+1)}& =a'_{(i,j,t)}+\tau \left[ \frac{1}{\gamma }\left( \alpha \left( 1-a'_{(i,j,t)}\right) -m'_{(i,j,t)}a'_{(i,j,t)}\right) \right] . \end{aligned} \end{array}\right. } \end{aligned}$$

We will use the following real data as well as estimated values:

**Table 1:** Parameter values used in the simulation

Symbol	Value	Unit	Source
*c*	0.1	$\textrm{m}^3$/g/h	Scholten and Smaal (1998), Riisgård (2001)
*e*	0.2	g/g	Sukhotin et al. (2002)
$d_M$	0.014	g/g/h	Estimated
$k_M$	200	$\mathrm{g/m}^2$	Estimated
$A_{\text {up}}$	1	$\mathrm{g/m}^3$	Cadée and Hegeman (2002)
*f*	100	$\textrm{m}^3$ $\mathrm{/m}^3$/h	Koppel et al. (2005)
*H*	0.1	m	Wildish and Kristmanson (1984)
			Muschenheim and Newell (1992)
*V*	360 (10)	m/h (cm/s)	Brinkman et al. (2002)
$D_M$	0.0112–0.112	$\textrm{m}^2$/h	Estimated
$D_{\bar{M}}$	1.4–1.68	$\textrm{m}^2$/h	Estimated
$D_{\bar{A}}$	$2.1\times 10^{-5}$–$4.9\times 10^{-5}$	$\textrm{m}^2$/h	Estimated
$D_A$	0.014–0.028	$\textrm{m}^2$/h	Estimated
$\tau$	250–614	h	Estimated
$\delta$	10–20	m	Estimated

It is worth noting that in Table 1, $\text {g}/\text {g}$ represents an organism gains 0.2 gs of body mass for every 1 g of food consumed and $\text {g}/\text {g}/\text {h}$ stands for the number of grams lost or gained per gram of organism per hour.

So, based on the nondimensionalization process we have discussed earlier:

$$\omega =200, r\approx 1.4, \alpha =0.5, \gamma =7\times 10^{-5},\tilde{\tau }=\tau =3.5\sim 8.6$$

and

$$\begin{aligned} d_{m_1}=0.8\sim 8, d_{m_2}=0.5\sim 0.6, d_{a_1}=0.3\sim 0.7, d_{a_2}=1\sim 2. \end{aligned}$$

Under the parameter conditions described above, we find that the system does not exhibit flip or Turing bifurcations. In fact, abrupt changes in external environmental conditions such as rising water temperature, decreasing salinity, and hypoxia (Bolotov et al. 2018; Smith et al. 2006; Maar et al. 2024), can lead to significant changes in the mussel’s mortality rate and saturation density. Therefore, in order to better simulate these phenomena, we will adopt larger values of $\gamma$ in the subsequent numerical simulations to reflect realistic conditions.

To regulate the dynamics of the system, this study proposes a PD control of the form:

6 $$\begin{aligned} U_{(i,j, t+1)}=k_{p}\left( a'_{(i,j, t)}-a^{ref}_{(i,j, t)}\right) +k_{d}\frac{\left( a_{(i,j, t+1)}-a'_{(i,j, t)}\right) }{\tau } \end{aligned}$$

for model (5), where $k_{p}$ and $k_{d}$ represent the proportional gain and the differential gain, respectively. Additionally, $a^{ref}_{(i,j, t)}$ stands for the target reference state or desired value of the controlled variable.

### Remark 1

The PD control parameters can be linked to concrete management practices in the mussel–algae system. The proportional ($k_p$) term represents instantaneous feedback to deviations in algal density. When algal biomass falls below the desired level, management actions may include reducing mussel grazing pressure or supplementing nutrient input; conversely, when algal biomass becomes excessive, overgrowth can be suppressed by increasing mussel density or limiting nutrient input. The derivative ($k_d$) term corresponds to an anticipatory response to the rate of change in algal density. For instance, when algal abundance declines too rapidly, interventions such as temporarily reducing mussel grazing intensity may contrast this decline; whereas when algal abundance rises sharply, early measures such as enhancing mussel grazing or restricting nutrient input can be taken to prevent the occurrence of algal blooms. This interpretation is consistent with adaptive management frameworks that emphasise feedback-based regulation in ecosystems (Maxwell et al. 2017; Pelletier et al. 2020), and also comply with early-warning approaches in aquatic systems, such as mussel behaviour–based biological monitoring (Pilbala et al. 2024) and predictive models for harmful algal blooms (Muhammad et al. 2024).

After incorporating the PD control (6) into (5), we arrive at the controlled M-A model with self and cross-diffusion that takes the form of

7 $$\begin{aligned} {\left\{ \begin{array}{ll} \begin{aligned} m^{\prime }(i, j, t)& =m(i, j, t)+\frac{\tau }{\delta ^2} d_{m_1} \nabla _d^2 m(i, j, t)+\frac{\tau }{\delta ^2} d_{m_2} \nabla _d^2 a(i, j, t),\\ a^{\prime }(i, j, t)& =a(i, j, t)+\frac{\tau }{\delta ^2} d_{a_1} \nabla _d^2 m(i, j, t)+\frac{\tau }{\delta ^2} d_{a_2} \nabla _d^2 a(i, j, t),\\ \nabla _d^2m_{(i,j,t)}& =m_{(i+1,j,t)}+m_{(i-1,j,t)}+m_{(i,j+1,t)}+m_{(i,j-1,t)}-4m_{(i,j,t)},\\ \nabla _d^2a_{(i,j,t)}& =a_{(i+1,j,t)}+a_{(i-1,j,t)}+a_{(i,j+1,t)}+a_{(i,j-1,t)}-4a_{(i,j,t)},\\ m_{(i,j,t+1)}& =m'_{(i,j,t)}+\tau \left[ r a'_{(i,j,t)}m'_{(i,j,t)}-\frac{m'_{(i,j,t)}}{1+m'_{(i,j,t)}}\right] ,\\ a_{(i,j,t+1)}& =a'_{(i,j,t)}+\frac{\tau }{1-k_d}\bigg [\frac{1}{\gamma }\left( \alpha \left( 1-a'_{(i,j,t)}\right) -m'_{(i,j,t)}a'_{(i,j,t)}\right) \bigg .\\ & ~~~+\bigg .k_p\left( a'_{(i,j, t)}-a^{ref}_{(i,j, t)}\right) \bigg ]. \end{aligned} \end{array}\right. } \end{aligned}$$

We consider that system (7) is subject to the following periodic boundary conditions, which allows for a more realistic simulation of the local dynamics in an infinitely large or boundary-free natural ecosystem:

8 $$\begin{aligned} \begin{aligned}&m_{(i,0,t)}=m_{(i,n,t)},\quad m_{(i,1,t)}=m_{(i,n+1,t)},\quad \\ &m_{(0,j,t)}=m_{(n,j,t)},\quad m_{(1,j,t)}=m_{(n+1,j,t)}, \\&a_{(i,0,t)}=a_{(i,n,t)},\quad a_{(i,1,t)}=a_{(i,n+1,t)},\quad a_{(0,j,t)}=a_{(n,j,t)},\quad a_{(1,j,t)}=a_{(n+1,j,t)}. \\ \end{aligned} \end{aligned}$$

All the parameters are positive regardless of $k_{p}$ and $k_{d}$, and $m_{(i,j,t)}\ge 0, a_{(i,j,t)}\ge 0.$ In the following, we investigate the dynamics of the controlled CMLs model (7) with the boundary conditions (8).

### Remark 2

When both control parameters $k_{p}$ and $k_{d}$ are zero, the controlled CMLs model (7) becomes the uncontrolled CMLs model. Therefore, in the following, except for the case of $k_{p}=0$ and $k_{d}=0$, we uniformly call equations (7) the controlled CMLs model (7).

The CMLs model exhibits both homogeneous and heterogeneous spatial behaviors. For all *i*, *j*, and *t*, the homogeneous behavior satisfies

9 $$\begin{aligned} \nabla _d^2m_{(i,j,t)}=0,\quad \nabla _d^2a_{(i,j,t)}=0. \end{aligned}$$

In conjunction with equations (7), the homogeneous dynamics, where the spatial site indices are neglected, are governed by:

10a $$\begin{aligned} m_{t+1}=m_{t}+\tau \left( rm_{t}a_{t}-\frac{m_{t}}{1+m_{t}}\right) , \end{aligned}$$

10b $$\begin{aligned} a_{t+1}=a_t+\frac{\tau }{1-k_{d}} \left[ \frac{1}{\gamma }(\alpha (1-a_t)-m_ta_t)+k_{p} (a_t-a_{*} )\right] . \end{aligned}$$

which is possible to represent also as:

11 $$\begin{aligned} \left( \begin{array}{l} m \\ a \end{array}\right) \mapsto \left( \begin{array}{l} m+\tau \left( rma-\frac{m}{1+m}\right) \\ a+\frac{\tau }{1-k_{d}} \left[ \frac{1}{\gamma }(\alpha (1-a)-ma)+k_{p} (a-a_{*} )\right] \end{array}\right) . \end{aligned}$$

By analysing map (11), we can derive the homogeneous dynamics of the controlled CMLs model (7). We also remark that in order to achieve heterogeneous behavior, there must be at least one set of *i*, *j*, and *t* such that $\nabla _{d}^{2}m_{(i,j,t)}\ne 0$ and $\nabla _{d}^{2}a_{(i,j,t)}\ne 0.$

### Remark 3

Clearly, when the control parameter $k_{d}$ and $k_{p}$ are both zero, map (11) has a unique uniform coexistence fixed point $(m_{*},a_{*})$, where

$$ m_* = \frac{\alpha (r-1)}{1-\alpha r}, \ a_*=\frac{\alpha }{\alpha +m_*} = \frac{1}{r(1+m_*)} = \frac{1-\alpha r}{r(1-\alpha )} $$

if either of the following conditions is satisfied:

$$\begin{aligned} (C_{1})~~0<\frac{1}{\alpha }<r<1, ~~~~~~~~ and ~~~~~~~~ (C_{2})~~0<\alpha<\frac{1}{r}<1. \end{aligned}$$

In this study, we regard $a_*=\frac{\alpha }{\alpha +m_*}$ as the target reference state $a^{ref}_{(i,j, t)}$, which is reasonable because the $a_{*}$ corresponds to the system’s long-term stable behavior. Moreover, PD control offers the following advantages: (1) the location of the fixed point remains unchanged, as the PD control vanishes at the steady state; (2) the local dynamical structure near the fixed point, including the Jacobian matrix and its eigenvalue distribution, is preserved, allowing the original bifurcation mechanisms to remain dominant; (3) the ecological interpretation of the variables and parameters is unaffected; and (4) the control term is introduced as an additive component without altering the original CMLs framework. Therefore, the PD control regulates flip and Turing bifurcations without changing the intrinsic dynamical characteristics of the system.

## The stability analysis of the homogeneous stationary state

As mentioned in Remark 3, the fixed point $(m_{*},a_{*})$ of map (11) can be determined by solving the following equations (12):

12 $$\begin{aligned} {\left\{ \begin{array}{ll}m=m+\tau \left( rma-\frac{m}{1+m}\right) ,\\ \\ a=a+\frac{\tau }{1-k_{d}}\left[ \frac{1}{\gamma }(\alpha (1-a)-ma)+k_{p} (a-a_{*} )\right] \end{array}\right. } \end{aligned}$$

and for the positive fixed point $(m_{*},a_{*})$, the corresponding Jacobian matrix is

13 $$\begin{aligned} J(\tau )=\left( \begin{array}{cc} 1-\frac{(r-1) \alpha (r \alpha -1) \tau }{(\alpha -1 )^2} & \frac{r \alpha ( 1-r) \tau }{r \alpha -1} \\ \frac{(r \alpha -1) \tau }{r (\alpha -1) \gamma (k_d-1)} & 1+\frac{(\gamma k_p +r \alpha (\alpha -1 -\gamma k_p )) \tau }{(r \alpha -1) \gamma (k_d-1)} \\ \end{array} \right) . \end{aligned}$$

Notice that the two eigenvalues are

$$ \lambda _{1,2}=\frac{1}{2}\left( -p(\tau )\pm \sqrt{p^2(\tau )-4q(\tau )}\right) , $$

where $$p(\tau )=-TrJ(\tau )$$, $$q(\tau )=DetJ(\tau )$$ and

$$\begin{aligned} TrJ(\tau )=2-\frac{(-1+r) \alpha (-1+r \alpha ) \tau }{(-1+\alpha )^2}+\frac{(\gamma k_p +r \alpha (-1+\alpha -\gamma k_p )) \tau }{(-1+r \alpha ) \gamma (-1+k_d )}, \end{aligned}$$

and

$$\begin{aligned} \begin{aligned} DetJ(\tau )=&\frac{ \alpha \tau \left( r (\alpha -1)^2-(r-1) (r \alpha -1)^2 \tau \right) }{(\alpha -1) (r \alpha -1) \gamma (k_d-1)}\\&+\frac{\left( (r-1) \alpha (r \alpha -1) \tau -(\alpha -1)^2\right) (1-k_d +k_p \tau )}{(\alpha -1)^2 (k_d -1)}. \end{aligned} \end{aligned}$$

Consider the following quantities:

$$\begin{aligned} k_{d}^{*}= &  1+\frac{(\alpha -1 )^3}{(r-1) \alpha (r \alpha -1)^2 \gamma }, \\ k_{p}^{*}= &  -2 \sqrt{\frac{(r-1) \alpha (k_{d}-1)}{(\alpha -1 ) \gamma }}\\ &  +\frac{\alpha \left( \gamma +r \left( (\alpha -1 )^3+(-1-(r-1) \alpha (r \alpha -2 )) \gamma \right) +(r-1) (r \alpha -1)^2 \gamma k_{d}\right) }{(\alpha -1)^2 (r \alpha -1) \gamma },\\ \tau _N= &  \frac{r^2 \alpha ^2 (2+\alpha ) \gamma (k_{d}-1)+\gamma (k_{p} +\alpha ((\alpha -2) k_{p} +k_{d}-1 ))-r^3 \alpha ^3 \gamma (k_{d}-1)}{(r-1) \alpha (r \alpha -1)^2 (\alpha -1 -\gamma k_{p} )}\\ &  +\frac{r \alpha \left( \alpha ^3-1+\gamma -\alpha ^2 (3+\gamma k_{p} )+\alpha (3+2 \gamma (1+k_{p} -k_{d} ))-\gamma (k_{p} +k_{d} )\right) }{(r-1) \alpha (r \alpha -1)^2 (\alpha -1 -\gamma k_{p} )},\\ \tau _f= &  \tau _N-\sqrt{\tau _N^2-\frac{4 (\alpha -1)^2 \gamma (1-k_{d} )}{(r-1) \alpha (r \alpha -1) (\alpha -1 -\gamma k_p)}},\\ \tau _{\bar{f}}= &  \tau _N+\sqrt{\tau _N^2-\frac{4 (\alpha -1)^2 \gamma (1-k_{d} )}{(r-1) \alpha (r \alpha -1) (\alpha -1 -\gamma k_{p} )}},\\ \gamma _0= &  -\frac{(\alpha -1)^3}{(r-1) \alpha (r \alpha -1)^2}. \end{aligned}$$

By Ref. Nayfeh and Balachandran (2008) and direct analysis, we obtain the following proposition concerning the stability of the fixed point $(m_{*},a_{*})$.

### Proposition 1

For the fixed point $(m_{*},a_{*})$, if condition $(C_2)$ in Remark 3 and

1. one of the conditions $(SD_1)$–$(SD_4)$ below hold, then the fixed point is a saddle, where
  $$\begin{aligned} &  (SD_1)~~ \left\{ \begin{aligned}&k^{*}_{d}<k_d<1,\\&k_{p}<\frac{\alpha -1}{\gamma },\\&0<\tau<\tau _{\bar{f}}; \end{aligned} \right. ~(SD_2)~~ \left\{ \begin{aligned}&k_d\le k^{*}_{d},\\&k_{p}<\frac{\alpha -1}{\gamma },\\&0<\tau<\tau _{\bar{f}}; \end{aligned} \right. ~(SD_3)~~ \left\{ \begin{aligned}&k_d>1,\\&k_{p}>\frac{\alpha -1}{\gamma },\\&0<\tau<\tau _{\bar{f}}; \end{aligned} \right. \\ &  ~(SD_4)~~ \left\{ \begin{aligned}&k^{*}_d<k_d<1,\\&\frac{\alpha -1}{\gamma }<k_{p}<k^{*}_{p},\\&\tau _{f}<\tau <\tau _{\bar{f}}; \end{aligned} \right. \end{aligned}$$
2. one of the conditions $(SN_1)$–$(SN_3)$ below are satisfied, then the fixed point is a stable node, where
  $$\begin{aligned} &  (SN_1)~~ \left\{ \begin{aligned}&0<k_{d}<1,\\&\gamma =\gamma _0,\\&\frac{\alpha -1}{\gamma }<k_{p}<k_{p}^{*},\\&0<\tau<\tau _f; \end{aligned} \right. ~~~~~~~~(SN_2)~~ \left\{ \begin{aligned}&k^{*}_{d}<k_{d}<1,\\&0<\gamma<\gamma _0,\\&\frac{\alpha -1}{\gamma }<k_{p}<k_{p}^{*},\\&0<\tau<\tau _f; \end{aligned} \right. ~~~~~~~~\\ &  (SN_3)~~ \left\{ \begin{aligned}&k^{*}_{d}<k_{d}<1,\\&\gamma >\gamma _0,\\&\frac{\alpha -1}{\gamma }<k_{p}<k_{p}^{*},\\&0<\tau <\tau _f; \end{aligned} \right. \end{aligned}$$
3. one of the conditions $(SDN_1)$–$(SDN_3)$ below hold, then the fixed point is a stable degenerate node, where
  $$\begin{aligned}(SDN_1)~~ \left\{ \begin{aligned}&0<k_{d}<1,\\&\gamma =\gamma _0,\\&k_{p}=k_{p}^{*},\\&0<\tau<\tau _N; \end{aligned} \right. ~~~~~~~~(SDN_2)~~ \left\{ \begin{aligned}&k^{*}_{d}<k_{d}<1,\\&0<\gamma<\gamma _0,\\&k_{p}=k_{p}^{*},\\&0<\tau<\tau _N; \end{aligned} \right. ~~~~~~~~(SDN_3)~~ \left\{ \begin{aligned}&k^{*}_{d}<k_{d}<1,\\&\gamma >\gamma _0,\\&k_{p}=k_{p}^{*},\\&0<\tau <\tau _N. \end{aligned} \right. \end{aligned}$$

### Proof

Since the proof of the results only involves the calculation of eigenvalues of the corresponding Jacobian, it is a standard procedure and can be easy to follow so we outline the proof here:

(1) If a fixed point is a saddle point, the two eigenvalues of the characteristic equation $\lambda ^2+p\lambda +q=0$ must be distinct real roots, that is $p^2-4q>0$, and $\lambda _1=-\frac{p}{2}-\frac{1}{2}\sqrt{p^2-4q}<-\frac{p}{2}+\frac{1}{2}\sqrt{p^2-4q}=\lambda _2,$ with one having an absolute value greater than 1 and the other less than 1. Accordingly, we obtain two sets of necessary and sufficient conditions:

$$ \left\{ \begin{aligned}&p^2-4q>0\\&-1<\lambda _{1}<1<\lambda _{2} \end{aligned} \right. ~~\text {or}~~ \left\{ \begin{aligned}&p^2-4q>0\\&\lambda _{1}<-1<\lambda _{2}<1, \end{aligned} \right. $$

that is

$$ \left\{ \begin{aligned}&p<0\\&p-1<q<-p-1 \end{aligned} \right. ~~\text {or}~~ \left\{ \begin{aligned}&p>0\\&p-1>q>-p-1. \end{aligned} \right. $$

(2) The fixed point is stable if all $|\lambda _{1,2}|<1$; the fixed point is unstable if at least one of $|\lambda _{1,2}|>1$. Making a transformation: $\lambda =\frac{\omega +1}{\omega -1}$, and substituting it into the above characteristic equation, we get $(1+p+q)\omega ^2+2\omega (1-q)+1-p+q=0,$ then $|\lambda |<1\Leftrightarrow \text {Re}~ \omega <0$. The fixed point is stable $\Leftrightarrow \text {Re}~ \omega <0$ $\Leftrightarrow$ $\omega _1+\omega _2<0, \omega _1\omega _2>0.$ Thus,

$$\begin{aligned} \left\{ \begin{aligned}&\omega _1+\omega _2=-\frac{2(1-q)}{1+p+q}<0\\&\omega _1\omega _2=\frac{1-p+q}{1+p+q}>0 \end{aligned} \right. \Leftrightarrow \left\{ \begin{aligned}&1-q>0\\&1+p+q>0\\&1-p+q>0 \end{aligned} \right. ~~\text {or}~~ \left\{ \begin{aligned}&1-q<0\\&1+p+q<0~~(\text {discard}).\\&1-p+q<0 \end{aligned} \right. \end{aligned}$$

Then we can get the conditions that the fixed point is stable. Furthermore,

- if $p^2-4q>0$, the fixed point is a stable node;
- if $p^2-4q=0$, the fixed point is a stable degenerate node;
- if $p^2-4q<0$, the fixed point is a stable focus.

Finally, with the help of Mathematica, we can determine expressions of *p*, *q* to get the final results. $\square$

## Bifurcation analysis of the homogeneous steady state

This section examines $\tau$ as the key bifurcation parameter, emphasising the bifurcation dynamics of the homogeneous steady state within the controlled CMLs framework (7), particularly focussing on flip and Turing bifurcations. Through a theoretical approach, we establish the conditions for bifurcation in the controlled CMLs system (7) and outline the parameter conditions conducive to spatial pattern development. These insights provide a theoretical foundation for controlling bifurcation events and pattern types using PD control, thereby effectively stabilising the population density of algae *a* and ensuring a consistent spatial distribution.

### Flip bifurcation

The loss of stability of the fixed point and the occurrence of a flip bifurcation result in the emergence of period−2 points. The flip bifurcation takes place when one eigenvalue of $J(\tau )$ is exactly $-1$, while the others have modulus less than 1 at the critical threshold, that is, the bifurcation parameter must satisfy one of the following constraints:

$$ (H_1)~~ \left\{ \begin{aligned}&0<k_{d}<1,\\&\gamma =\gamma _0,\\&\frac{\alpha -1}{\gamma }<k_{p}<k_{p}^{*},\\&\tau =\tau _f,\\&\tau \ne \frac{2 (\alpha -1 )^2 (r \alpha -1 ) \gamma (k_d-1) }{(r-1)\alpha (r\alpha -1)^2\gamma (k_d-1)-\left( \gamma k_p+r\alpha (\alpha -1-\gamma k_p)\right) (\alpha -1)^2 }\\&~~~~~~\text {or}~ \frac{4 (\alpha -1 )^2 (r \alpha -1 ) \gamma (k_d-1) }{(r-1)\alpha (r\alpha -1)^2\gamma (k_d-1)-\left( \gamma k_p+r\alpha (\alpha -1-\gamma k_p)\right) (\alpha -1)^2 }; \end{aligned} \right. $$

$$ (H_2)~~ \left\{ \begin{aligned}&k^{*}_{d}<k_{d}<1,\\&0<\gamma<\gamma _0,\\&\frac{\alpha -1}{\gamma }<k_{p}<k_{p}^{*},\\&\tau =\tau _f,\\&\tau \ne \frac{2 (\alpha -1 )^2 (r \alpha -1 ) \gamma (k_d-1) }{(r-1)\alpha (r\alpha -1)^2\gamma (k_d-1)-\left( \gamma k_p+r\alpha (\alpha -1-\gamma k_p)\right) (\alpha -1)^2 }\\&~~~~~~\text {or}~ \frac{4 (\alpha -1 )^2 (r \alpha -1 ) \gamma (k_d-1) }{(r-1)\alpha (r\alpha -1)^2\gamma (k_d-1)-\left( \gamma k_p+r\alpha (\alpha -1-\gamma k_p)\right) (\alpha -1)^2 }; \end{aligned} \right. $$

$$ (H_3)~~ \left\{ \begin{aligned}&k^{*}_{d}<k_{d}<1,\\&\gamma >\gamma _0,\\&\frac{\alpha -1}{\gamma }<k_{p}<k_{p}^{*},\\&\tau =\tau _f,\\&\tau \ne \frac{2 (\alpha -1 )^2 (r \alpha -1 ) \gamma (k_d-1) }{(r-1)\alpha (r\alpha -1)^2\gamma (k_d-1)-\left( \gamma k_p+r\alpha (\alpha -1-\gamma k_p)\right) (\alpha -1)^2 }\\&~~~~~~\text {or}~ \frac{4 (\alpha -1 )^2 (r \alpha -1 ) \gamma (k_d-1) }{(r-1)\alpha (r\alpha -1)^2\gamma (k_d-1)-\left( \gamma k_p+r\alpha (\alpha -1-\gamma k_p)\right) (\alpha -1)^2 }. \end{aligned} \right. $$

Next, we employ the centre manifold theorem to reduce the map (11) while treating $\tau$ as the independent variable. We define $w=m-m_{*},$ $z=a-a_{*},$ and $\tilde{\tau }=\tau -\tau _{f}$. As a result, map (11) is transformed into the following form:

14 $$\begin{aligned} \left( \begin{array}{l} w \\ z \\ \tilde{\tau } \end{array}\right) =\left( \begin{array}{lll} a_{100} & a_{010} & 0 \\ b_{100} & b_{010} & 0 \\ 0 & 0 & 1 \end{array}\right) \left( \begin{array}{l} w \\ z \\ \tilde{\tau } \end{array}\right) +\left( \begin{array}{l} f_1(w, z, \tilde{\tau }) \\ f_2(w, z, \tilde{\tau }) \\ f_3(w, z, \tilde{\tau }) \end{array}\right) \end{aligned}$$

where

$$\begin{aligned} \begin{aligned} f_1(w, z, \tilde{\tau })&=a_{200}w^2+a_{110} w z+a_{101} w \tilde{\tau } +a_{011}z \tilde{\tau }+a_{201}w^2 \tilde{\tau }\\ &\quad + a_{111}w z \tilde{\tau }+a_{300} w^3+O(4),\\ f_2(w, z, \tilde{\tau })&=b_{110}w z+b_{101}w \tilde{\tau } +b_{011}z \tilde{\tau }+ b_{111}w z \tilde{\tau } +O(4),\\ f_3(w, z, \tilde{\tau })&=0, \end{aligned} \end{aligned}$$

and $a_{100}= 1-\frac{(r-1) \alpha (r \alpha -1 ) \tau _f }{(\alpha -1 )^2}$, $a_{010}=\frac{r \alpha (1 -r) \tau _f }{r \alpha -1 }$, $a_{200}=\frac{(r \alpha -1 )^3 \tau _f}{(\alpha -1 )^3}$, $a_{110}=r\tau _f$, $a_{101}=-\frac{(r-1) \alpha (r\alpha -1)}{(\alpha -1)^2}$, $a_{011}=\frac{r\alpha (1 -r)}{r\alpha -1}$, $a_{201}=\frac{(r \alpha -1 )^3}{(\alpha -1)^3}$, $a_{111}=r$, $a_{300}=-\frac{(r \alpha -1)^4 \tau _f}{(\alpha -1 )^4}$; $b_{100}=\frac{(r \alpha -1) \tau _f }{r (\alpha -1) \gamma (k_{d}-1) },$ $b_{010}=1+\frac{(\gamma k_p +r \alpha (\alpha -1 -\gamma k_p )) \tau _f }{(r \alpha -1) \gamma (k_d-1)}$, $b_{110}=-\frac{\tau _f}{\gamma (1-k_d )}$, $b_{101}=-\frac{(r \alpha -1 )}{r (\alpha -1 ) \gamma (1-k_d )}$, $b_{011}=\frac{\gamma k_p +r \alpha (\alpha -1 -\gamma k_p )}{(r \alpha -1 ) \gamma (k_d-1)}$, $b_{111}=-\frac{1}{\gamma (1-k_{d} ) }$, and *O*(4) represent polynomial terms in the variables $(w,z,\tau )$ of order greater than or equal to 4.

Subsequently, performing the inverse transformation $w=a_{020}(\tilde{w}+\tilde{z})$ and $z=(-1-a_{100})\tilde{w}+(\lambda _2-a_{100})\tilde{z}$ with $\lambda _2=1+a_{100}+b_{010},$ system (14) can be converted to the following expression:

15 $$\begin{aligned} \begin{aligned}&\tilde{w} \mapsto -\tilde{w}+\frac{1}{a_{010}\left( 1+\lambda _2\right) } F_1(\tilde{w}, \tilde{z}, \tilde{\tau }), \\&\tilde{z} \mapsto \lambda _2 \tilde{z}+\frac{1}{a_{010}\left( 1+\lambda _2\right) } F_2(\tilde{w}, \tilde{z}, \tilde{\tau }), \\&\tilde{\tau } \mapsto \tilde{\tau }, \end{aligned} \end{aligned}$$

here

$$\begin{aligned} \begin{aligned}&F_1(\tilde{w}, \tilde{z},\tilde{\tau })\\ &\quad =\left( \lambda _2-a_{100}\right) \Big [(\tilde{w}+\tilde{z}) \tilde{\tau } a_{010} a_{101}+(\tilde{w}+\tilde{z})^2 a_{010}^2 a_{200}+(\tilde{w}+\tilde{z})^2 \tilde{\tau } a_{010}^2 a_{201}\Big .\\&\qquad \Big .+(\tilde{w}+\tilde{z})^3 a_{010}^3 a_{300}+\tilde{\tau } a_{011} \left( \tilde{w} \left( -1-a_{100}\right) +\tilde{z} \left( -a_{100}+\lambda _2\right) \right) \Big .\\&\qquad \Big .+(\tilde{w}+\tilde{z}) a_{010} a_{110} \left( \tilde{w} \left( -1-a_{100}\right) +\tilde{z} \left( -a_{100}+\lambda _2\right) \right) \Big .\\&\qquad \Big .+(\tilde{w}+\tilde{z}) \tilde{\tau } a_{010} a_{111} \left( \tilde{w} \left( -1-a_{100}\right) +\tilde{z} \left( -a_{100}+\lambda _2\right) \right) \Big ]\\&\qquad -a_{010} \Big [(\tilde{w}+\tilde{z}) \tilde{\tau } a_{010} b_{101}+\tilde{\tau } b_{011} \left( \tilde{w} \left( -1-a_{100}\right) +\tilde{z} \left( -a_{100}+\lambda _2\right) \right) \Big .\\&\qquad \Big .+(\tilde{w}+\tilde{z}) a_{010} b_{110} \left( \tilde{w} \left( -1-a_{100}\right) +\tilde{z} \left( -a_{100}+\lambda _2\right) \right) +(\tilde{w}+\tilde{z}) \tilde{\tau } a_{010}\Big .\\&\qquad \Big .\times b_{111} \left( \tilde{w} \left( -1-a_{100}\right) +\tilde{z} \left( -a_{100}+\lambda _2\right) \right) \Big ] + O(4), \end{aligned} \end{aligned}$$

and

$$\begin{aligned} \begin{aligned}&F_2(\tilde{w},\tilde{z},\tilde{\tau })\\ &\quad = \left( 1+a_{100}\right) \Big [(\tilde{w}+\tilde{z}) \tilde{\tau } a_{010} a_{101}+(\tilde{w}+\tilde{z})^2 a_{010}^2 a_{200}+(\tilde{w}+\tilde{z})^2 \tilde{\tau } a_{010}^2 a_{201}\Big .\\&\qquad \Big .+(\tilde{w}+\tilde{z})^3 a_{010}^3 a_{300}+\tilde{\tau } a_{011} \left( \tilde{w} \left( -1-a_{100}\right) +\tilde{z} \left( -a_{100}+\lambda _2\right) \right) \Big .\\&\qquad \Big .+(\tilde{w}+\tilde{z}) a_{010} a_{110} \left( \tilde{w} \left( -1-a_{100}\right) +\tilde{z} \left( -a_{100}+\lambda _2\right) \right) \Big .\\&\qquad \Big .+(\tilde{w}+\tilde{z}) \tilde{\tau } a_{010} a_{111} \left( \tilde{w} \left( -1-a_{100}\right) +\tilde{z} \left( -a_{100}+\lambda _2\right) \right) \Big ]\\&\qquad +a_{010} \Big [(\tilde{w}+\tilde{z}) \tilde{\tau } a_{010} b_{101}+\tilde{\tau } b_{011} \left( \tilde{w} \left( -1-a_{100}\right) +\tilde{z} \left( -a_{100}+\lambda _2\right) \right) \Big .\\&\qquad \Big .+(\tilde{w}+\tilde{z}) a_{010} b_{110} \left( \tilde{w} \left( -1-a_{100}\right) +\tilde{z} \left( -a_{100}+\lambda _2\right) \right) \Big .\\&\qquad \Big .+(\tilde{w}+\tilde{z}) \tilde{\tau } a_{010} b_{111} \left( \tilde{w} \left( -1-a_{100}\right) +\tilde{z} \left( -a_{100}+\lambda _2\right) \right) \Big ] + O(4). \end{aligned} \end{aligned}$$

To gain insight into the stability of the bifurcated period-2 orbit, it is essential to derive the governing equation based on the centre manifold. We proceed with the assumption that the centre manifold is represented as follows:

16 $$\begin{aligned} W^c(0,0,0)=\left\{ (\tilde{w}, \tilde{z}, \tilde{\tau }) \in R^3 \mid \tilde{z}=h^*(\tilde{w}, \tilde{\tau }), h^*(0,0)=0, D h^*(0,0)=0\right\} , \end{aligned}$$

where $h^*(\tilde{w}, \tilde{\tau })=e_1 \tilde{w}^2+e_2 \tilde{w} \tilde{\tau }+e_3 \tilde{\tau }^2+O(3).$ By substituting $\tilde{z}=h^*(\tilde{w},\tilde{\tau })$ into map (15), one can derive that

17 $$\begin{aligned} \begin{aligned}&\lambda _2h^*(w,\tilde{\tau })+\frac{F_2(\tilde{w},h^*(\tilde{w},\tilde{\tau }),\tilde{\tau })}{a_{010}(1+\lambda _2)}\\ &\quad =e_1\left[ -\tilde{w}+\frac{F_1(\tilde{w},h^*(\tilde{w},\tilde{\tau }),\tilde{\tau })}{a_{010}(1+\lambda _2)}\right] ^2\\ &\qquad +e_2\left[ -\tilde{w}+\frac{F_1(\tilde{w},h^*(\tilde{w},\tilde{\tau }),\tau )}{a_{010}(1+\lambda _2)}\right] \tilde{\tau }+e_3\tilde{\tau }^2+O(3). \end{aligned} \end{aligned}$$

By comparing the terms $\tilde{w}^2$, $\tilde{w}\tilde{\tau }$, $\tilde{\tau }^2,$ we can obtain the following results

18 $$\begin{aligned} \begin{aligned}&e_1=\frac{\left( 1+a_{100}\right) \left( \left( 1+a_{100}\right) a_{110}+a_{010} \left( b_{110}-a_{200}\right) \right) }{-1+\lambda _2^2},\\&e_2=\frac{a_{011} \left( 1+a_{100}\right) {}^2-a_{010} \left( \left( 1+a_{100}\right) a_{101}-\left( 1+a_{100}\right) b_{011}+a_{010} b_{101}\right) }{a_{010} \left( 1+\lambda _2\right) {}^2},\\&e_3=0. \end{aligned} \end{aligned}$$

By restricting the dynamics of the map (15) to the centre manifold, it is possible to achieve

19 $$\begin{aligned} F: \tilde{w}\mapsto -\tilde{w}+\mu _{1}\tilde{w}^{2}+\mu _{2}\tilde{w}\tilde{\tau }+\mu _{3}\tilde{w}^{2}\tilde{\tau }+\mu _{4}\tilde{w}\tilde{\tau }^{2}+\mu _{5}\tilde{w}^{3}+O(4), \end{aligned}$$

here

$$\begin{aligned} \mu _{1}&=\frac{a_{100}^2 a_{110}+a_{100} \left( a_{010} \left( b_{110}-a_{200}\right) -a_{110} \left( \lambda _2-1\right) \right) -a_{110} \lambda _2+a_{010} \left( b_{110}+a_{200} \lambda _2\right) }{1+\lambda _2}, \\ \mu _{2}&=\frac{-a_{010} \left( a_{010} b_{101}-\left( 1+a_{100}\right) b_{011}\right) +\left( a_{011} \left( 1+a_{100}\right) -a_{010} a_{101}\right) \left( a_{100}-\lambda _2\right) }{a_{010} \left( 1+\lambda _2\right) }, \\ \mu _3&=\frac{1}{a_{010} \left( 1+\lambda _2\right) }\Big [a_{011} e_1 \left( a_{100}-\lambda _2\right) {}^2+a_{010} \left( a_{100}-\lambda _2\right) \left( \left( 1+a_{100}\right) a_{111}-a_{101} e_1\right. \Big .\\&\quad \Big .\left. +b_{011} e_1+a_{110} e_2+2 a_{100} a_{110} e_2-a_{110} e_2 \lambda _2\right) +a_{010}^2 \left( b_{111}-b_{101} e_1+b_{110} e_2\right. \Big .\\&\quad \Big .\left. +a_{100} \left( b_{111}-a_{201}+2 \left( b_{110}-a_{200}\right) e_2\right) +a_{201} \lambda _2+2 a_{200} e_2 \lambda _2-b_{110} e_2 \lambda _2\right) \Big ],\\ \mu _{4}&=\frac{e_2 \left( a_{011} \left( a_{100}-\lambda _2\right) {}^2-a_{010} \left( a_{100} \left( a_{101}-b_{011}\right) +a_{010} b_{101}+\left( b_{011}-a_{101}\right) \lambda _2\right) \right) }{a_{010} \left( 1+\lambda _2\right) },\\ \mu _5&=\frac{1}{1+\lambda _2}\left[ a_{010}^2 a_{300} \left( \lambda _2-a_{100}\right) +a_{010} e_1 \left( -2 a_{100} \left( a_{200}-b_{110}\right) -b_{110} \left( \lambda _2-1\right) \right. \right. \\&\quad \left. \left. +2 a_{200} \lambda _2\right) +a_{110} e_1 \left( 2 a_{100}^2+a_{100} \left( 1-3 \lambda _2\right) +\left( \lambda _2-1\right) \lambda _2\right) \right] . \end{aligned}$$

According to the flip bifurcation theorem (Guckenheimer and Holmes 2013), the occurrence of a flip bifurcation in the map (19) requires the term below to be different than zero:

$$\begin{aligned} \begin{aligned} \eta _1&=\left( \frac{\partial ^2F}{\partial \tilde{w}\partial \tilde{\tau }}+\frac{1}{2}\frac{\partial F}{\partial \tilde{\tau }}\cdot \frac{\partial ^2F}{\partial \tilde{w}^2}\right) \Bigg |_{(\tilde{w},\tilde{\tau })=(0,0)}=\mu _2,\\ \eta _2&=\left( \frac{1}{6}\frac{\partial ^3F}{\partial \tilde{w}^3}+\left( \frac{1}{2}\frac{\partial ^2F}{\partial \tilde{w}^2}\right) ^2\right) \Bigg |_{(\tilde{w},\tilde{\tau })=(0,0)}=\mu _5+\mu _1^2, \end{aligned} \end{aligned}$$

which indeed is the case for our system, thus allowing us to reach the following conclusion.

#### Theorem 2

The controlled CMLs model (7) undergoes a flip bifurcation at $(m_{*},a_{*})$, provided that the following conditions are satisfied:

- ($$C_2$$) holds,
- one of the conditions ($H_1$)–($H_3$) is satisfied, and
- $$\eta _1\eta _2\ne 0.$$

Moreover, if $\eta _2 >0$, a stable periodic−2 orbit bifurcates from $(m_{*},a_{*})$; and if $\eta _2<0$, an unstable periodic−2 orbit bifurcate from $(m_{*},a_{*})$.

### Turing bifurcation

Turing bifurcation is primarily induced by the breaking of spatial symmetry. When Turing instability occurs, the initially stable homogeneous stationary state of the controlled cellular automaton model (7) becomes unstable due to discrepancies in spatial diffusion, leading to the emergence of spatial patterns. According to Proposition 1, if condition ($C_2$) and one of the conditions ($SN_1$)–($SN_3$), ($SDN_1$)–($SDN_3$) are satisfied, then $(m_{*},a_{*})$ will remain stable over time, and so, in this section, we examine the Turing bifurcation of the homogeneous stationary state.

To determine the conditions that induce Turing instability, we solve the eigenvalue problem related to the discrete Laplacian operator $\nabla _d^2$. It is well known that, for a discrete Laplacian operator $\nabla _d^2$, the eigenvalue $\lambda$ can be derived by solving the following equation:

20 $$\begin{aligned} \nabla _d^2X^{ij}+\lambda X^{ij}=0, i, j=1,2,\cdots ,n, \end{aligned}$$

and fulfill the following periodic boundary conditions:

21 $$\begin{aligned} X^{i,0}=X^{i,n},X^{i,1}=X^{i,n+1},X^{0,j}=X^{n,j},X^{1,j}=X^{n+1,j}. \end{aligned}$$

So (see for example Ref. Bai and Zhang (2009)), the eigenvalues of the discrete Laplacian operator $\nabla _{d}^{2}$ are:

22 $$\begin{aligned} \lambda _{kl}=4\bigg (\sin ^2\bigg (\frac{(k-1)\pi }{n}\bigg )+\sin ^2\bigg (\frac{(l-1)\pi }{n}\bigg )\bigg ),~l, k\in \{1,2,\cdots ,n\}. \end{aligned}$$

In order to investigate the Turing bifurcation, a spatially inhomogeneous perturbation is introduced to the spatially uniform stationary state $(m_{*},a_{*})$. The equation expressing this spatially inhomogeneous perturbation for the system at hand is given by:

23 $$\begin{aligned} \tilde{m}_{(i,j,t)}=m_{(i,j,t)}-m_{*},\tilde{a}_{(i,j,t)}=a_{(i,j,t)}-a_{*}, \end{aligned}$$

and we note that $\nabla _d^2\tilde{m}_{(i,j,t)}=\nabla _d^2m_{(i,j,t)},\nabla _d^2\tilde{a}_{(i,j,t)}=\nabla _d^2a_{(i,j,t)},$ and the values of the two are not always zero.

By substituting the above perturbation equation into the controlled CMLs model equation, we obtain:

24a $$\begin{aligned} \begin{aligned} \tilde{m}_{(i,j,t+1)}&=a_{10}\left( \tilde{m}_{(i,j,t)}+\frac{\tau }{\delta ^2}d_{m_1}\nabla ^2_d\tilde{m}_{(i,j,t)}+\frac{\tau }{\delta ^2}d_{m_2}\nabla ^2_d\tilde{a}_{(i,j,t)}\right) \\&\quad +a_{01}\left( \tilde{a}_{(i,j,t)}+\frac{\tau }{\delta ^2}d_{a_1}\nabla _d^2\tilde{m}_{(i,j,t)}+\frac{\tau }{\delta ^2}d_{a_2}\nabla _d^2\tilde{a}_{(i,j,t)}\right) \\&\quad +O\left( \left( \left| \tilde{m}_{(i,j,t)}\right| +\left| \tilde{a}_{(i,j,t)}\right| \right) ^2\right) , \end{aligned} \end{aligned}$$

24b $$\begin{aligned} \begin{aligned} \tilde{a}_{(i,j,t+1)}&= b_{10}\left( \tilde{m}_{(i,j,t)}+\frac{\tau }{\delta ^2}d_{m_1}\nabla ^2_d\tilde{m}_{(i,j,t)}+\frac{\tau }{\delta ^2}d_{m_2}\nabla ^2_d\tilde{a}_{(i,j,t)}\right) \\&\quad +b_{01}\left( \tilde{a}_{(i,j,t)}+\frac{\tau }{\delta ^2}d_{a_1}\nabla _d^2\tilde{m}_{(i,j,t)}+\frac{\tau }{\delta ^2}d_{a_2}\nabla _d^2\tilde{a}_{(i,j,t)}\right) \\&\quad +O\left( \left( \left| \tilde{m}_{(i,j,t)}\right| +\left| \tilde{a}_{(i,j,t)}\right| \right) ^2\right) . \end{aligned} \end{aligned}$$

When the perturbation is small, the linear terms govern the dynamics of system (24). By multiplying both sides of equation (24) with the eigenvector $X_{kl}^{ij}$ corresponding to the eigenvalue $\lambda _{kl}$, we arrive at:

25a $$\begin{aligned} \begin{aligned}X_{kl}^{ij}\tilde{m}_{(i,j,t+1)}&=a_{10}X_{kl}^{ij}\tilde{m}_{(i,j,t)}+a_{01}X_{kl}^{ij}\tilde{a}_{(i,j,t)}\\&\quad +\frac{\tau }{\delta ^2}\left( a_{10}d_{m_1}+a_{01}d_{a_1}\right) X_{kl}^{ij}\nabla _{d}^{2}\tilde{m}_{(i,j,t)}\\&\quad +\frac{\tau }{\delta ^2}\left( a_{10}d_{m_2}+a_{01}d_{a_2}\right) X_{kl}^{ij}\nabla _{d}^{2}\tilde{a}_{(i,j,t)}, \end{aligned} \end{aligned}$$

25b $$\begin{aligned} \begin{aligned}X_{kl}^{ij}\tilde{a}_{(i,j,t+1)}&=b_{10}X_{kl}^{ij}\tilde{m}_{(i,j,t)}+b_{01}X_{kl}^{ij}\tilde{a}_{(i,j,t)}\\&\quad +\frac{\tau }{\delta ^2}\left( b_{10}d_{m_1}+b_{01}d_{a_1}\right) X_{kl}^{ij}\nabla _{d}^{2}\tilde{m}_{(i,j,t)}\\&\quad +\frac{\tau }{\delta ^2}\left( b_{10}d_{m_2}+b_{01}d_{a_2}\right) X_{kl}^{ij}\nabla _{d}^{2}\tilde{a}_{(i,j,t)}. \end{aligned} \end{aligned}$$

Taking the sum over all *i* and *j* on both sides of equation (25) yields:

26a $$\begin{aligned} \begin{aligned}\sum _{i,j=1}^{n} X_{kl}^{ij}\tilde{m}_{(i,j,t+1)}&=a_{10}\sum _{i,j=1}^{n}X_{kl}^{ij}\tilde{m}_{(i,j,t)}+a_{01}\sum _{i,j=1}^{n}X_{kl}^{ij}\tilde{a}_{(i,j,t)})\\&\quad +\frac{\tau }{\delta ^2}\left( a_{10}d_{m_1}+a_{01}d_{a_1}\right) \\&\quad \times \sum _{i,j=1}^{n}X_{kl}^{ij}\nabla _{d}^{2}\tilde{m}_{(i,j,t)})\\&\quad +\frac{\tau }{\delta ^2}\left( a_{10}d_{m_2}+a_{01}d_{a_2}\right) \sum _{i,j=1}^{n}X_{kl}^{ij}\nabla _{d}^{2}\tilde{a}_{(i,j,t)}, \end{aligned} \end{aligned}$$

26b $$\begin{aligned} \begin{aligned}\sum _{i,j=1}^{n}X_{kl}^{ij}\tilde{a}_{(i,j,t+1)}&=b_{10}\sum _{i,j=1}^{n}X_{kl}^{ij}\tilde{m}_{(i,j,t)}+b_{01}\sum _{i,j=1}^{n}X_{kl}^{ij}\tilde{a}_{(i,j,t)}\\&\quad +\frac{\tau }{\delta ^2}\left( b_{10}d_{m_1}+b_{01}d_{a_1}\right) \\&\quad \times \sum _{i,j=1}^{n}X_{kl}^{ij}\nabla _{d}^{2}\tilde{m}_{(i,j,t)}\\&\quad +\frac{\tau }{\delta ^2}\left( b_{10}d_{m_2}+b_{01}d_{a_2}\right) \sum _{i,j=1}^{n}X_{kl}^{ij}\nabla _{d}^{2}\tilde{a}_{(i,j,t)}. \end{aligned} \end{aligned}$$

Let $\overline{m}_t=\sum _{i,j=1}^{n} X_{kl}^{ij}\tilde{m}_{(i,j,t)},\overline{a}_t=\sum _{i,j=1}^{n} X_{kl}^{ij}\tilde{a}_{(i,j,t)},$ then system (26) can be transformed into the following form:

27a $$\begin{aligned} \begin{aligned}\overline{m}_{t+1}=A_{11}\overline{m}_t+A_{12}\overline{a}_t\end{aligned}, \end{aligned}$$

27b $$\begin{aligned} \begin{aligned}\overline{a}_{t+1}=A_{21}\overline{m}_t+A_{22}\overline{a}_t\end{aligned}, \end{aligned}$$

where

$$\begin{aligned} A_{11}= &  a_{10}-\frac{\tau }{\delta ^2}\left( a_{10}d_{m_1}+a_{01}d_{a_1}\right) \lambda _{kl},A_{12}=a_{01}-\frac{\tau }{\delta ^2}\left( a_{10}d_{m_2}+a_{01}d_{a_2}\right) \lambda _{kl}, \\ A_{21}= &  b_{10}-\frac{\tau }{\delta ^2}\left( b_{10}d_{m_1}+b_{01}d_{a_1}\right) \lambda _{kl},A_{22}=b_{01}-\frac{\tau }{\delta ^2}\left( b_{10}d_{m_2}+b_{01}d_{a_2}\right) \lambda _{kl}. \end{aligned}$$

The dynamic behavior of the spatially inhomogeneous perturbation solutions is described by system (27). When the system of equations diverges, the discrete system will experience spatial symmetry breaking at $(m_{*},a_{*})$, resulting in the emergence of Turing patterns. Obviously, the divergence of the discrete system of equations is directly associated with the two eigenvalues:

28 $$\begin{aligned} \lambda _\pm (k,l)=\frac{1}{2}\bigg (\big (A_{11}+A_{22}\big )\pm \sqrt{\big (A_{11}+A_{22}\big )^2-4A_{12}A_{21}}\bigg ), \end{aligned}$$

when $|\lambda _{+}(k,l)|>1$ or $|\lambda _{-}(k,l)|>1$, the fixed point (0, 0) of system (27) is unstable, indicating that the homogeneous stationary state $(m_{*},a_{*})$ becomes unstable.

Moreover, let us denote

29 $$\begin{aligned} Z(k,l,\tau )=\max \left\{ |\lambda _+(k,l)|,|\lambda _-(k,l)|\right\} , \end{aligned}$$

30 $$\begin{aligned} Z_m(\tau )=\max \limits _{k=1}^n\max \limits _{l=1}^n Z(k,l,\tau ),(k,l)\ne (1,1). \end{aligned}$$

The threshold condition for Turing bifurcation is given by $Z_m(\tau ')=1,$ and the critical value $\tau '$ is given by the following proposition.

#### Proposition 3

1. When $\tau$ is close to $\tau '$, if $\left( A_{11}(k,l,\tau ')+A_{22}(k,l,\tau ')\right) ^2>4A_{12}(k,l,\tau ')A_{21}(k,l,\tau ')$ is satisfied, then the critical value $\tau '$ can be determined by $\max \limits _{k=1,l=1}^n\left( \big |A_{11}(k,l,\tau ')+A_{22}(k,l,\tau ')\big |-A_{12}(k,l,\tau ')A_{21}(k,l,\tau ')\right) =1$.
2. If $(A_{11}(k,l,\tau ')+A_{22}(k,l,\tau '))^2\le 4A_{12}(k,l,\tau ')A_{21}(k,l,\tau ')$ for $\tau$ sufficiently close to $\tau '$, then the critical value $\tau '$ is determined by $\max \limits _{k=1,l=1}^n A_{12}(k,l,\tau ')A_{21}(k,l,\tau ')=1$.

Building on the above analysis, we present the following theorems.

#### Theorem 4

Under the assumption that condition ($C_2$) and one of conditions ($SN_1$)–($SN_3$), ($SDN_1$)–($SDN_3$) is valid, and $\tau$ is close to $\tau '$. If $Z_m(\tau )>1,$ then the homogeneous stationary state $(m_{*},a_{*})$ of the controlled CMLs model (7) with periodic conditions (8) is subject to Turing instability, leading to the emergence of Turing patterns. Conversely, if $Z_m(\tau )<1,$ the homogeneous stationary state $(m_{*},a_{*})$ of the controlled CMLs model (7) remains stable, and no Turing pattern arise.

#### Remark 4

Theorem 4 primarily characterises the occurrence of Turing bifurcation, where $Z_m(\tau )$ serves as a critical criterion. On a set of $n \times n$ lattice sites, each point (*k*, *l*) corresponds to a pair of eigenvalues $\lambda _{\pm }(k,l)$. Therefore, there are $2n^2$ eigenvalues in total. Under the assumption of spatially uniform steady-state stability, when the parameter approaches the critical threshold $\tau '$, if the modulus of every pair of eigenvalues at each node remains less than 1, the controlled CMLs model (7) will remain stable, without Turing instability or Turing pattern formation. However, if there exist eigenvalues at certain sites with a modulus greater than 1, the controlled CMLs model (7) exhibits Turing instability, leading to the emergence of Turing patterns. According to equations (28)–(30), $Z_m(\tau )$ measures the extent to which the modulus of these $2n^2$ eigenvalues is greater than or less than 1, thereby serving as an indicator to determine whether Turing instability occurs as $\tau$ approaches its critical threshold $\tau '$.

## Numerical simulation

This section presents several numerical simulations to demonstrate various dynamic behaviors of the controlled CMLs model (7), including flip and Turing bifurcations. The corresponding spatiotemporal patterns are also analyzed. Furthermore, the effectiveness of applying a PD control to regulate algae population density and maintain a uniform distribution is validated.

### Simulations for flip bifurcation

First, we examine the temporal dynamics associated with the flip bifurcation, which corresponds to the results established in Theorem 2.

**Fig. 2:**
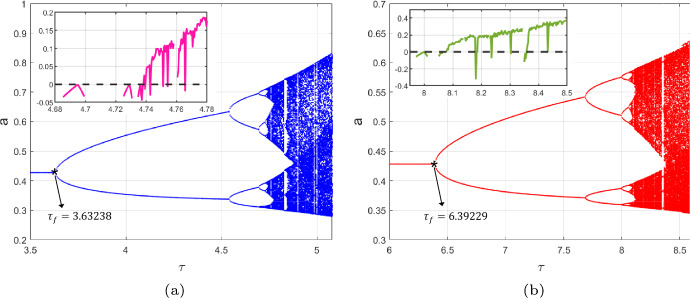
The flip bifurcation and maximum Lyapunov exponent diagram for uncontrolled systems with $k_{p}=0$, $k_{d}=0$ and controlled systems with $k_{p}=-0.1$, $k_{d}=-0.5$

We set $\alpha =0.5$, $r=1.4$, $\gamma =1.2$, which means that condition ($C_2$) is satisfied. Initially, we examine the uncontrolled system with $k_{p}=0$, $k_{d}=0$. The system has a unique positive fixed point at $(m_{*},a_{*})=(0.6667,0.4286)$, and the critical value $\tau _f=3.63238$. Setting $\tau =\tau _f$, the eigenvalues are −1 and 0.340291, confirming that condition ($H_2$) is satisfied. Furthermore, our calculations yield $\eta _1=-0.550603<0$ and $\eta _2=153.347>0$. According to Theorem 2, the uncontrolled CMLs model (7) undergoes a flip bifurcation at $(m_{*}, a_{*})$ when $\tau =\tau _f$, leading to the emergence of a stable period−2 orbit bifurcating from $(m_{*}, a_{*})$. This implies that the population density of algae *a* becomes unstable at $\tau =\tau _f$. To illustrate this, we present the flip bifurcation diagram of the algae population density over the monitoring time $\tau$; see Fig. 2a. When considering the controlled CMLs model (7), we set $k_{d}=-0.5$, $k_{p}=-0.1$, while keeping the values of $\alpha$, *r* and $\gamma$ unchanged. The corresponding flip bifurcation diagram of the population density of algae *a* is depicted in Fig. 2b. Upon analyzing the two figures in Fig. 2, it is evident that the population density of algae *a* exhibits periodic doubling as the monitoring time $\tau$ varies. By comparison, it is clear that the population density of algae *a* in Fig. 2b fluctuates later than that in Fig. 2a. Furthermore, by plotting the graph of the maximum Lyapunov exponent, we observe that when no control is applied, the population density of *a* enters a chaotic state around $\tau =4.72$. However, after implementing the control, the population density of *a* becomes chaotic around $\tau =8.1$, which means that the introduction of PD control can effectively delay the occurrence of chaos.

**Fig. 3:**
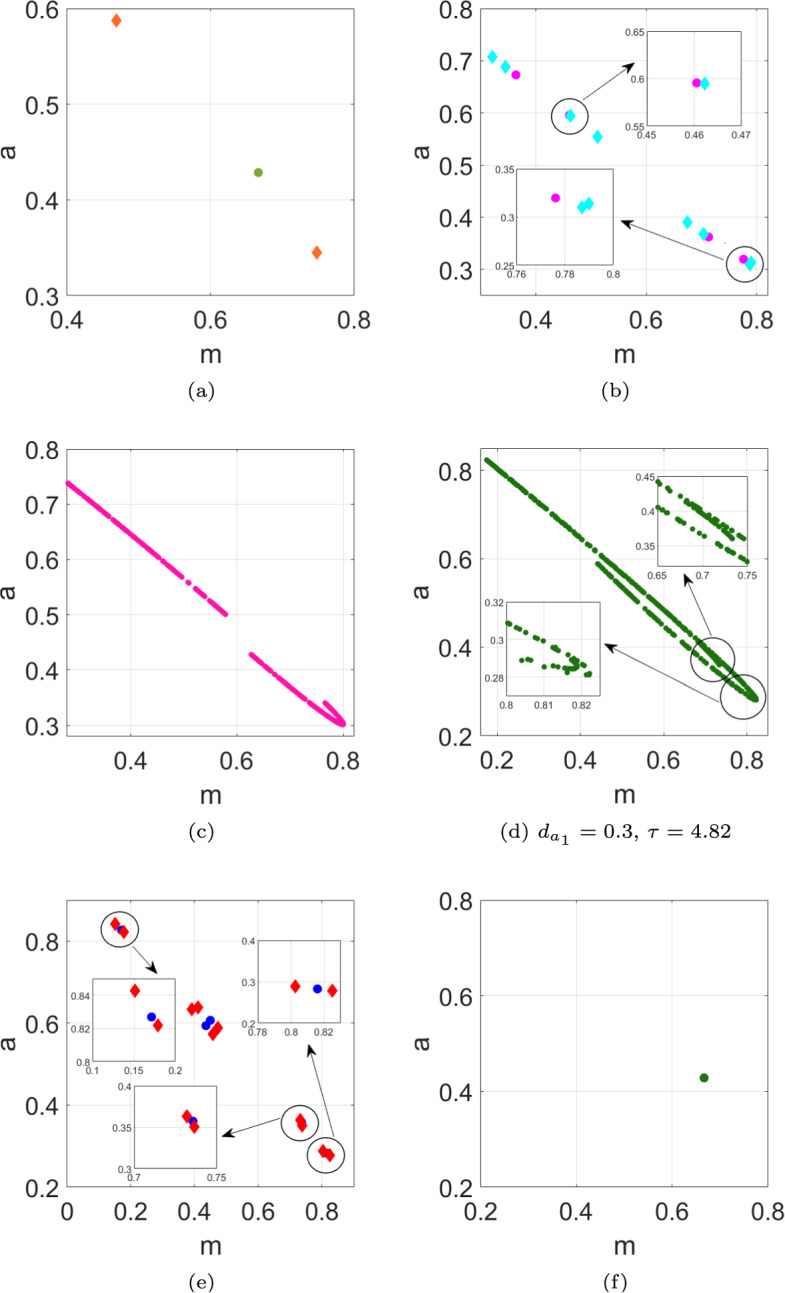
Phase portraits for different values of $\tau$. **a** The light green dot represents a stable node at $\tau =3.6$, orange diamonds represent the period−2 points at $\tau =4.2$; **b** the magenta dots (cyan diamonds) stand for the period−4 (period−8) points at $\tau =4.6$ ($\tau =4.72$); **c** a distinct periodic orbit at $\tau =4.82$; **d** chaos occurs at $\tau =5.06$; **e** the blue dots (red diamonds) stand for the period−5 (period−10) points at $\tau =5.1$ ($\tau =5.15$); **f** a stable node at $\tau =5.06$ with $k_p=-0.1$, $k_d=-0.5$

In the following, we investigate the dynamic transition of map (11) with increasing monitoring time $\tau$, which includes the occurrence of a stable node at $\tau =3.6$, the period−2 orbit when $\tau =4.2$, please refer to Fig. 3a. Additionally, period−4, 8, 5 and 10 orbit are observed at $\tau =4.6$, 4.72, 5.1 and 5.15, respectively, as illustrated in Fig. 3b and e. This may suggest that the population density of mussel *m* or algae *a* exhibit fluctuations in four, eight, five and ten distinct states. In Fig. 3c, a distinct periodic orbit emerges at $\tau =4.82$. At this time, it is not possible to determine how the densities of the two populations fluctuate. In Fig. 3d, chaos occurs at $\tau =5.06$, indicating that the population density fluctuations of population mussel *m* or algae *a* are highly complex and irregular. With the application of PD control, it is observed that at $\tau = 5.06$, the population densities of both species stabilise, shifting from the previously chaotic state, as depicted in Figs. 3(f).

**Fig. 4:**
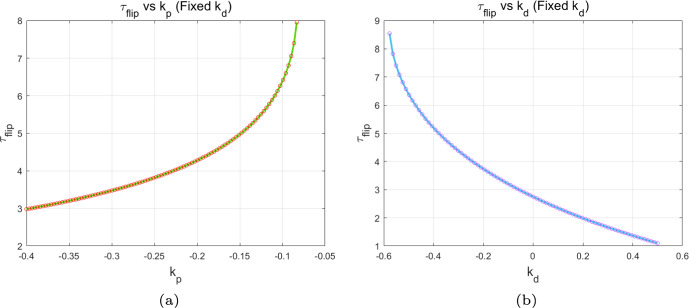
Variation of flip bifurcation threshold $\tau _f$ with respect to control parameters $k_{p}$ and $k_{d}$

Based on the above analysis, it can be concluded that the PD control effectively mitigates the multi-periodic fluctuations in algae population density and delays the emergence of chaos. To further elucidate the effect of the PD control, we investigate the relationship between the flip bifurcation threshold $\tau _f$ and the control parameters $k_{p}$ and $k_{d}$. When the two control parameters function independently, Figs. 4(a) and (b) depict the trend of the flip bifurcation threshold $\tau _f$ with respect to each of the control parameters $k_p$ and $k_d$, respectively. In summary, with $k_{d} = -0.5$ fixed, the flip bifurcation threshold increases as $k_{p}$ increases. This suggests that increasing $k_{p}$ helps delay the occurrence of the flip bifurcation, thereby postponing the onset of chaos in algae *a*. Conversely, with $k_{p} = -0.1$ fixed, the flip bifurcation threshold decreases as $k_{d}$ increases. This suggests that reducing $k_{d}$ effectively delays the flip bifurcation, thereby postponing the chaotic state of algae *a*.

### Simulations for Turing bifurcation and spatiotemporal patterns

In this part, we present a Turing bifurcation diagram to identify the critical value for Turing bifurcations and generate the corresponding Turing patterns based on the different regions of instability. Simultaneously, we will discuss the effectiveness of the PD control in adjusting Turing instability and shaping the resulting Turing patterns for different cases.

- Case 1: $$\alpha =0.5$$, $$r=1.4$$, $$\gamma =1.2$$ and $$d_{m_2}=0$$, $$d_{a_1}=0$$, $$\delta =10$$.

**Fig. 5:**
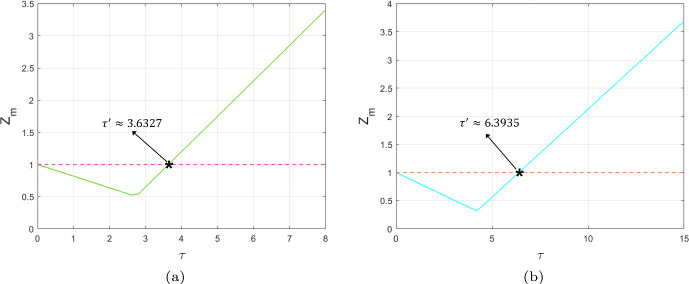
The $Z_m$–$\tau$ diagram indicates the existence of Turing instability for uncontrolled systems with $k_{p}=0$, $k_{d}=0$ and controlled systems with $k_{p}=-0.1$, $k_{d}=-0.5$. The intersection with $Z_m = 1$ indicates the threshold $\tau '$ of the Turing bifurcation

**Fig. 6:**
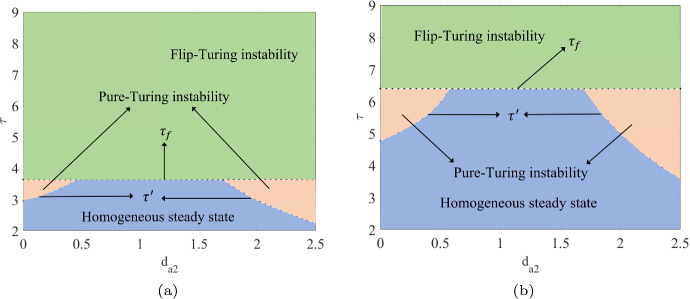
The $\tau -d_{a_2}$ plane is divided into three distinct regions, including homogeneous steady state, pure-Turing instability and flip-Turing instability for uncontrolled systems with $k_{p}=0$, $k_{d}=0$ and controlled systems with $k_{p}=-0.1$, $k_{d}=-0.5$, respectively

The occurrence of Turing instability is governed by the parameter $Z_m$, which in turn is determined by the maximum value of the function *Z*(*k*, *l*) evaluated over all modes $(k,l)\ne (1,1)$. Set $d_{m_1}=0.8$ and $d_{a_2}=1$. We plot the $Z_m$–$\tau$ diagram, which are displayed in Figs. 5. We observe that when $k_{p} = 0$ and $k_{d} = 0$, corresponding to the uncontrolled CMLs model (7), the Turing bifurcation threshold is $\tau ' \approx 3.6327$. However, when $k_{p} = -0.1$ and $k_{d} = -0.5$, corresponding to the controlled CMLs model (7), the Turing bifurcation threshold increases to $\tau ' \approx 6.3935$. Thus, the introduction of the control effectively delays the occurrence of the Turing bifurcation. In Figs. 6(a) and (b), we show the pattern formation regions for both the uncontrolled and controlled systems by combining the flip bifurcation curve, $\tau _f$, with the Turing bifurcation curve, $\tau '$, respectively. We have identified three distinct regions: the homogeneous stationary state region, the pure-Turing instability region, and the flip-Turing instability region. Within the same coordinate value range, it is evident that the Turing instability region of the controlled system, with $k_{p} = -0.1$ and $k_{d} = -0.5$, is noticeably smaller than that of the uncontrolled system, where $k_{p} = 0$ and $k_{d} = 0$. This indicates that the introduction of the control effectively regulates the Turing instability region, helping to stabilise the population density and ensuring a uniform distribution of algae *a*.

**Fig. 7:**
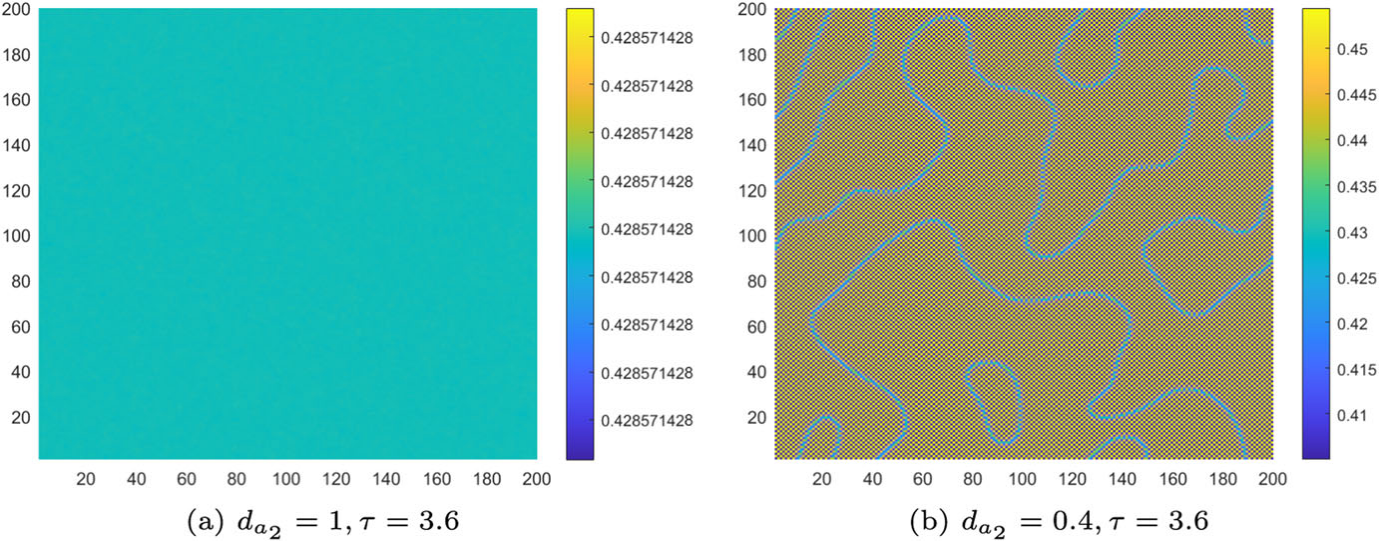
For uncontrolled systems with $k_{p}=0$, $k_{d}=0$, **a** the stable uniform steady state corresponds to an even distribution of algae *a* across space; **b** the pure-self-diffusion-Turing instability mechanism induces Turing patterns in algae, implying that the distribution of algae *a* becomes non-uniform

We also simulate the pattern evolution of both the controlled and uncontrolled CMLs model (7) for different monitoring time values $\tau$. Let $d_{a_2}=1$, $\tau =3.6$. From Fig. 6a, we know that the uncontrolled CMLs model (7) will neither undergo a flip bifurcation nor a Turing bifurcation. It remains in a stable homogeneous stationary state. At this point, no Turing patterns emerge. As shown in Fig. 7a, the algae *a* remains uniformly distributed in space. Now, we set $d_{a_2}=0.4$ and $\tau =3.6$. As depicted in Fig. 6a, the uncontrolled CMLs model (7) enters the pure-Turing instability region, indicating that it will only undergo the Turing bifurcation. The patterns are shown in Fig. 7b. We can see a mosaic pattern appear, which implies that the distribution of algae *a* is not uniform at this time.

**Fig. 8:**
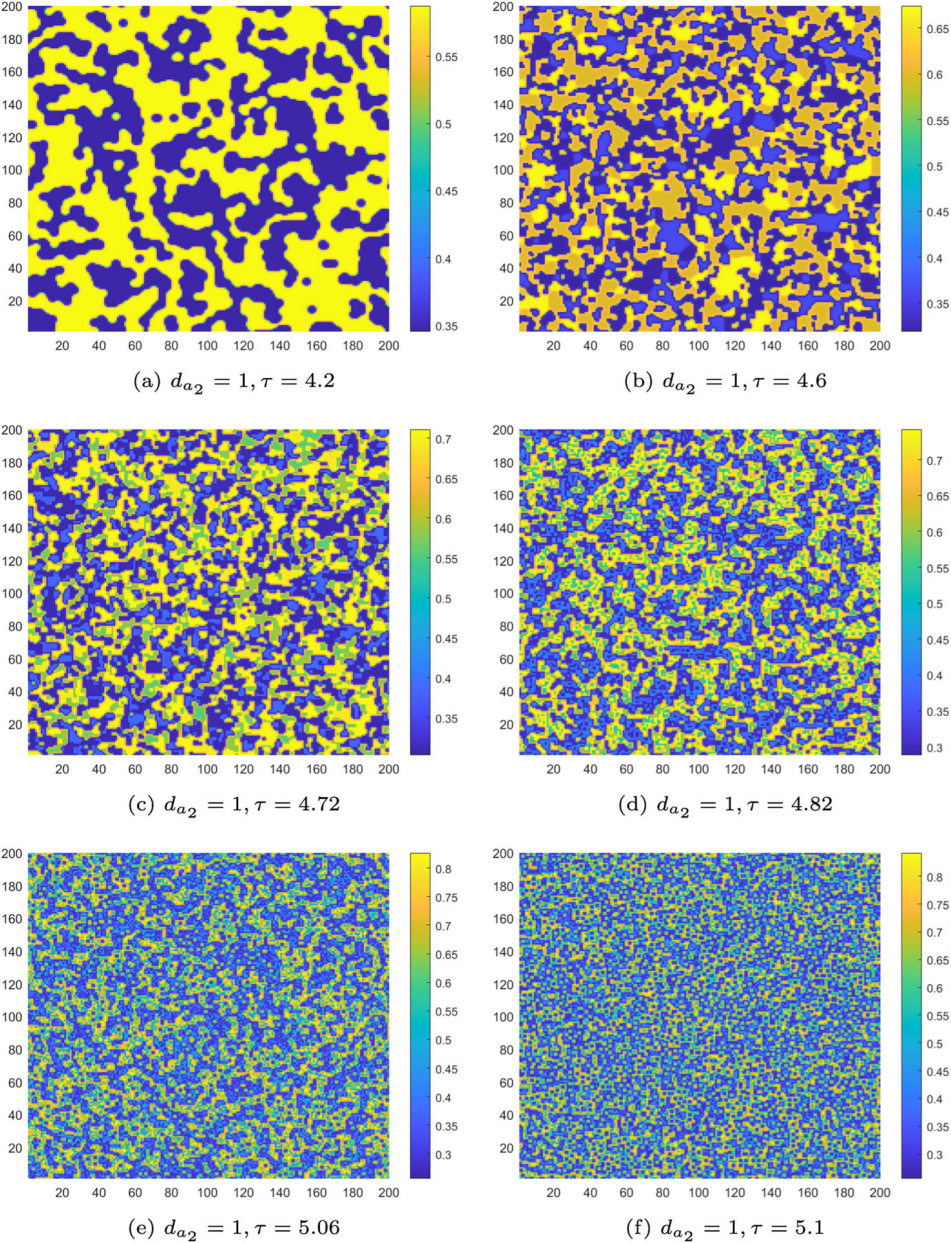
The Turing patterns of the algae *a* induced by the flip-self-diffusion-Turing instability mechanism at different $\tau$ values for uncontrolled systems with $k_{p}=0$, $k_{d}=0$

**Fig. 9:**
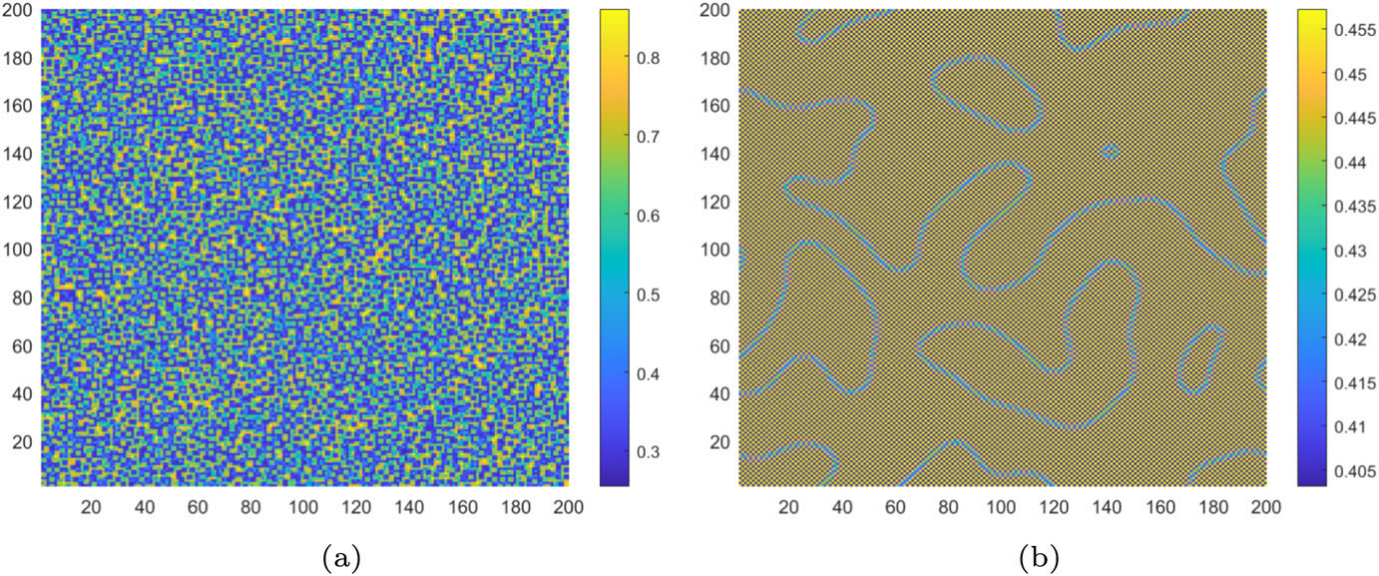
For uncontrolled systems with $k_{p}=0$, $k_{d}=0$, **a** the Turing pattern of algae *a* induced by the flip-self-diffusion-Turing instability mechanism at $\tau =5.15$; **b** the Turing pattern of algae *a* induced by the pure-cross-diffusion-Turing instability mechanism with $d_{m_1}=0.8$, $d_{m_2}=0.5$, $d_{a_1}=0.7$ and $d_{a_2}=1$ at $\tau =3.6$

Next, we adjust $\tau$ to place the homogeneous stationary state of the uncontrolled CMLs model (7) in different stable states and present the corresponding Turing patterns. Based on the phase portraits in Figs. 3, we set $d_{a_2} = 1$ and vary $\tau$ to the values 3.6, 4.2, 4.6, 4.72, 4.82, 5.06, 5.1 and 5.15. The pattern transformations of algae *a* in the uncontrolled CMLs model (7) during the period−doubling process are shown in Figs. 8(a)-(f) and 9(a).

For $\tau _f< \tau ' < \tau = 4.2$, the homogeneous stationary state becomes a stable period−2 orbit. The uncontrolled CMLs model (7) simultaneously undergoes both Turing and flip bifurcation, leading to the emergence of spatially heterogeneous patterns driven by the flip-Turing instability mechanism (see Fig. 8a). The spatial distribution of the algae *a* is primarily characterized by two alternating states. For $\tau =4.6$, we can observe that four distinct states are intertwined, as shown in Fig. 8b. For $\tau =4.72$, a more fragmented spatial pattern comprising eight interwoven states is observed (Fig. 8c), with period−8 orbit primarily governing the structure. Similarly, at $\tau = 5.1$ and $\tau = 5.15$, patterns consisting of five and ten states governed by the period−5 and period−10 orbits are observed, as shown in Figs. 8(f) and 9(a). We can also observe additional complex patterns, such as the one at $\tau = 4.82$, as shown in Fig. 8d. In particular, at $\tau = 5.06$, chaos arises, and the resulting patterns are fragmented and disordered, as illustrated in Fig. 8e. In conclusion, as the monitoring time $\tau$ increases, the patterns become increasingly complex, driven by the flip-self-diffusion-Turing instability mechanism.

However, the generation of the above discussed complex patterns also implies the uneven distribution of algae *a*, which can result in extreme behaviours for local ecosystems. Therefore, it is essential to discuss control to stabilise the M-A system. For $\tau =5.15$, choosing the control parameters $k_{p}=-0.1$, $k_{d}=-0.5$, the pattern transitions from a complex state to a stable state identical to what is shown in Fig. 7a. This indicates that implementing the control strategy is highly effective in regulating the pattern type.

It is important to note that all the Turing patterns described above are formed under the influence of self-diffusion. When $d_{a_2} = 1$ and $\tau = 3.6$, as shown in Figs. 6(a) and 7(a), the uncontrolled CMLs model exhibits a uniform spatial distribution. It is now interesting to introduce cross-diffusion. For instance, when $d_{m_{2}} = 0.5$ and $d_{a_{1}} = 0.7$, the uncontrolled CMLs model undergoes Turing instability, and the corresponding Turing patterns are shown in Fig. 9b. The patterns are driven by the pure-cross-diffusion-Turing instability mechanism. By observing Fig. 9b, we can find that the pattern type generated are largely the same as those produced by the pure-self-diffusion Turing instability mechanism as shown in Fig. 7b. Furthermore, under the influence of PD control (with parameters $k_{p} = -0.1$ and $k_{d} = -0.5$), the complex pattern can ultimately revert to a uniform distribution, as illustrated in Fig. 7a.

- Case 2: $$\alpha =0.5$$, $$r=1.4$$, $$\gamma =1.2$$ and $$d_{m_1}=0$$, $$d_{a_2}=0$$, $$\delta =20$$.

**Fig. 10:**
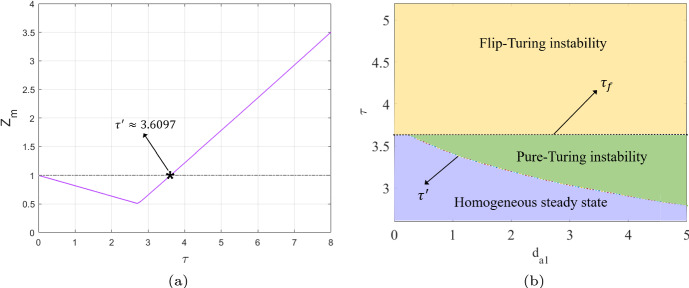
For the uncontrolled systems with $k_{p}=0$, $k_{d}=0$, **a** the $Z_m$–$\tau$ diagram indicates the existence of Turing instability; **b** the Turing instability regions indicate the different pattern formation mechanisms

Set $d_{m_2}=0.6$, $d_{a_1}=4$. Similarly to the previous case, we first present the $Z_m$–$\tau$ diagram in Fig. 10a, where we observe that the uncontrolled CMLs model undergoes Turing instability at $\tau ' \approx 3.6097$. Next, by varying $d_{a_1}$ from 0 to 5, we illustrate the Turing instability regions in Fig. 10b.

**Fig. 11:**
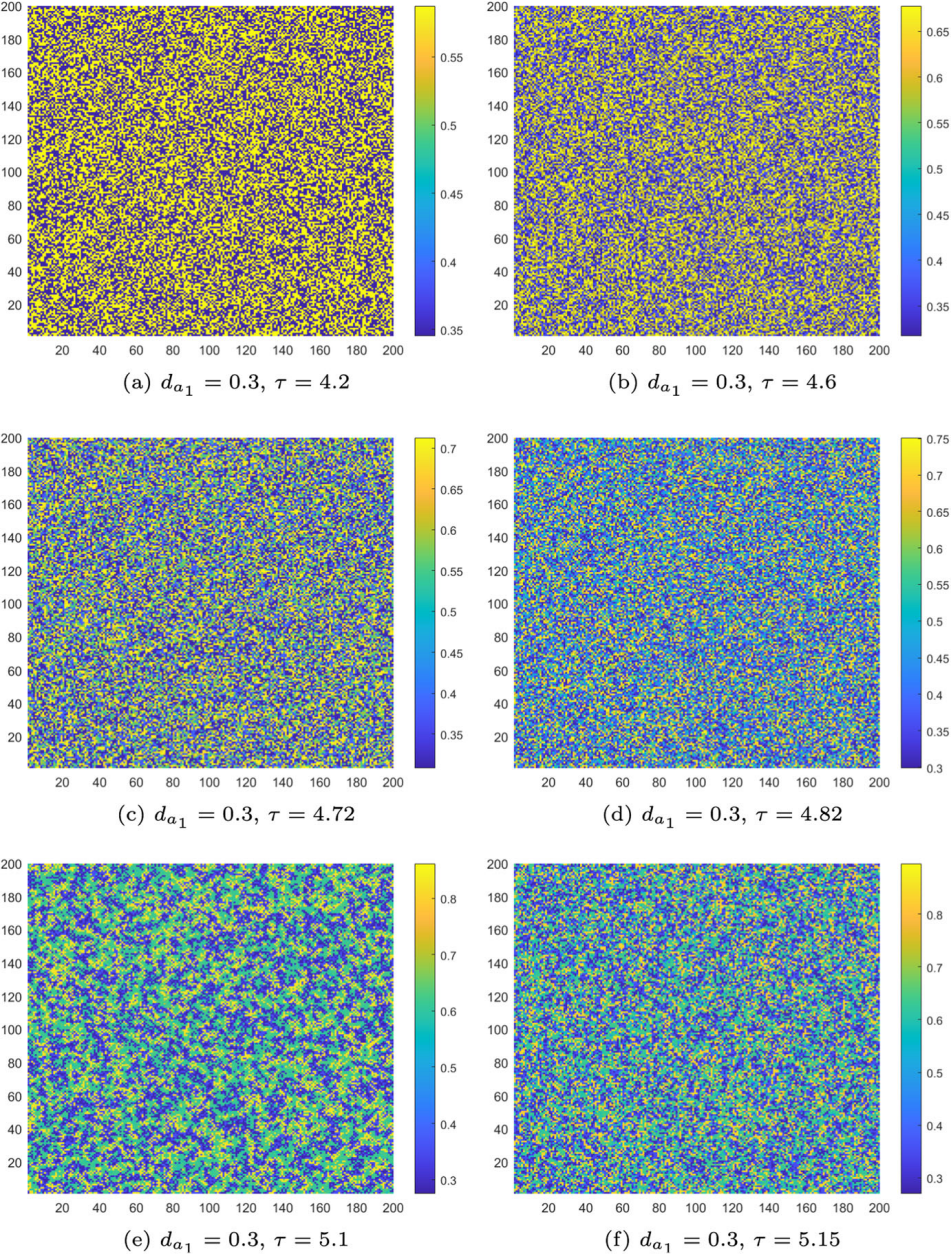
The Turing patterns of the algae *a* induced by the flip-cross-diffusion-Turing instability mechanism at different $\tau$ values for uncontrolled systems with $k_{p}=0$, $k_{d}=0$

In the following, numerical simulations are conducted to demonstrate the Turing patterns arising from different mechanisms. Let $d_{m_2}=0.6$, $d_{a_1}=0.3$. When $\tau = 3.6$, according to Fig. 10b, the CMLs model will neither undergo a flip bifurcation nor a Turing bifurcation. thus no Turing patterns are induced. The density of algae *a* is uniformly distributed in space like what is shown in Fig. 7a. When $\tau =4.2$, $\tau >\tau _f$ and $\tau >\tau '$; it means that the CMLs model can undergo both flip bifurcation and Turing bifurcation. The associated Turing patterns are shown in Fig. 11a. Similarly, the patterns consist of two alternating states, corresponding to the period−2 orbit that bifurcates from the fixed point through a flip bifurcation. Furthermore, when $\tau =4.6$ and 4.72, we can see that the patches of the resulting patterns are gradually breaking, see Fig. 11b and c. When $\tau =4.82$, the homogeneous stationary state becomes a complex periodic orbit, resulting in patterns that are more fragmented compared to the others, as depicted in Fig. 11d. As the value of $\tau$ continues to increase, such as $\tau =5.1$ and 5.15, the patterns with 5−state transitions and 10−state transitions induced by the period−5 and period−10 orbit appear, which are illustrated in Figs. 11(e) and (f). All these Turing patterns shown in Figs. 11 are induced by the flip-cross-diffusion-Turing instability mechanism. Similarly, this also implies that the CMLs model can undergo Turing instability in the presence of cross-diffusion alone, thereby leading to an uneven distribution of algae *a*. Interestingly, under the implementation of PD control, the unevenly distributed algae *a* at $\tau =4.82$ can also return to the uniform distribution state. Therefore, the PD control is also very effective for adjusting the Turing patterns induced by flip-cross-diffusion-Turing instability mechanism, so as to ensure a uniform and stable distribution of algae *a*.

**Fig. 12:**
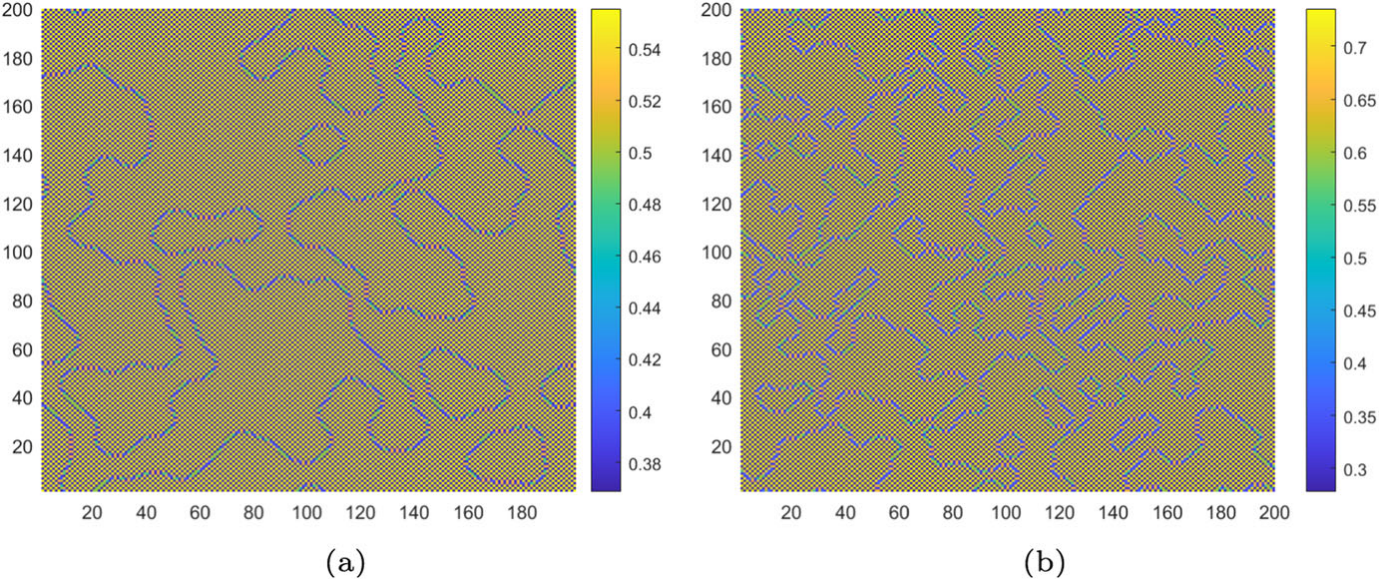
For uncontrolled systems ($k_{p}=0$, $k_{d}=0$), **a** the Turing pattern of algae *a* induced by pure-cross-diffusion-Turing instability mechanism with $d_{m_2}=0.6$, $d_{a_1}=4$ at $\tau =3.2$; **b** the Turing pattern of algae *a* induced by pure-self-diffusion-Turing instability mechanism with $d_{m_1}=8$, $d_{m_2}=0.6$, $d_{a_1}=0.3$ and $d_{a_2}=2$ at $\tau =3.6$

Then, we depict the Turing patterns resulting from the pure-cross-diffusion Turing instability and the pure-self-diffusion Turing instability mechanism, as shown in Fig. 12a and b. These patterns are also diverse and complex, resembling mosaic-like structures. Compared to the pure-self-diffusion Turing instability and the pure-cross-diffusion Turing instability mechanism of case 1 as presented in Fig. 7b and 9b, the colors of the patterns generated in Fig. 12a and b are more fragmented. We also introduce PD control to change it from a complex uneven distribution state to a uniform distribution, as presented in Fig. 7a.

**Fig. 13:**
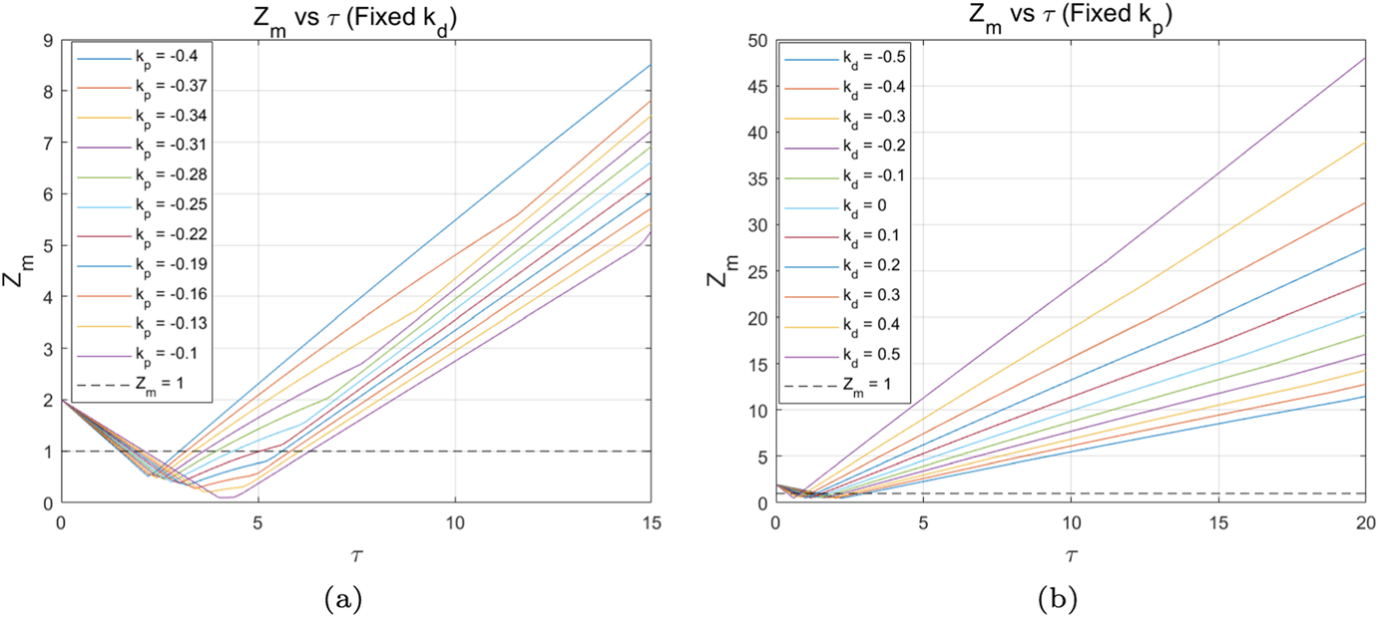
The $Z_m$–$\tau$ diagram for different values of $k_p$ with the control parameter $k_d=-0.5$; (b) the $Z_m$–$\tau$ diagram for different values of $k_d$ with the control parameter $k_p=-0.1$

In the following, we study the effect of PD control on the critical threshold of Turing bifurcation in CMLs model (7). By observing Fig. 13, when the two control parameters work independently, if $k_{d}$ is fixed, it is found that the Turing bifurcation threshold increases as $k_{p}$ increases. This means that increasing the value of $k_{p}$ is effectively delaying the occurrence of Turing bifurcation, which induces stability in the M-A system. When $k_{p}$ is fixed, it can be found that the Turing bifurcation threshold increases with the decrease of $k_{d}$, that is, the smaller $k_{d}$ is, the more uniform distribution of M-A system can be ensured, which maintains stability.

At the end of this section, we take the Turing patterns induced by flip-self-diffusion-Turing instability mechanism at $\tau =5.15$ as an example to demonstrate the variations in the patterns under different control parameters. In this process, only the control parameters $k_p$ and $k_d$ are varied, while all other parameters remain the same as in Figs. 8 and 9(a).

**Fig. 14:**
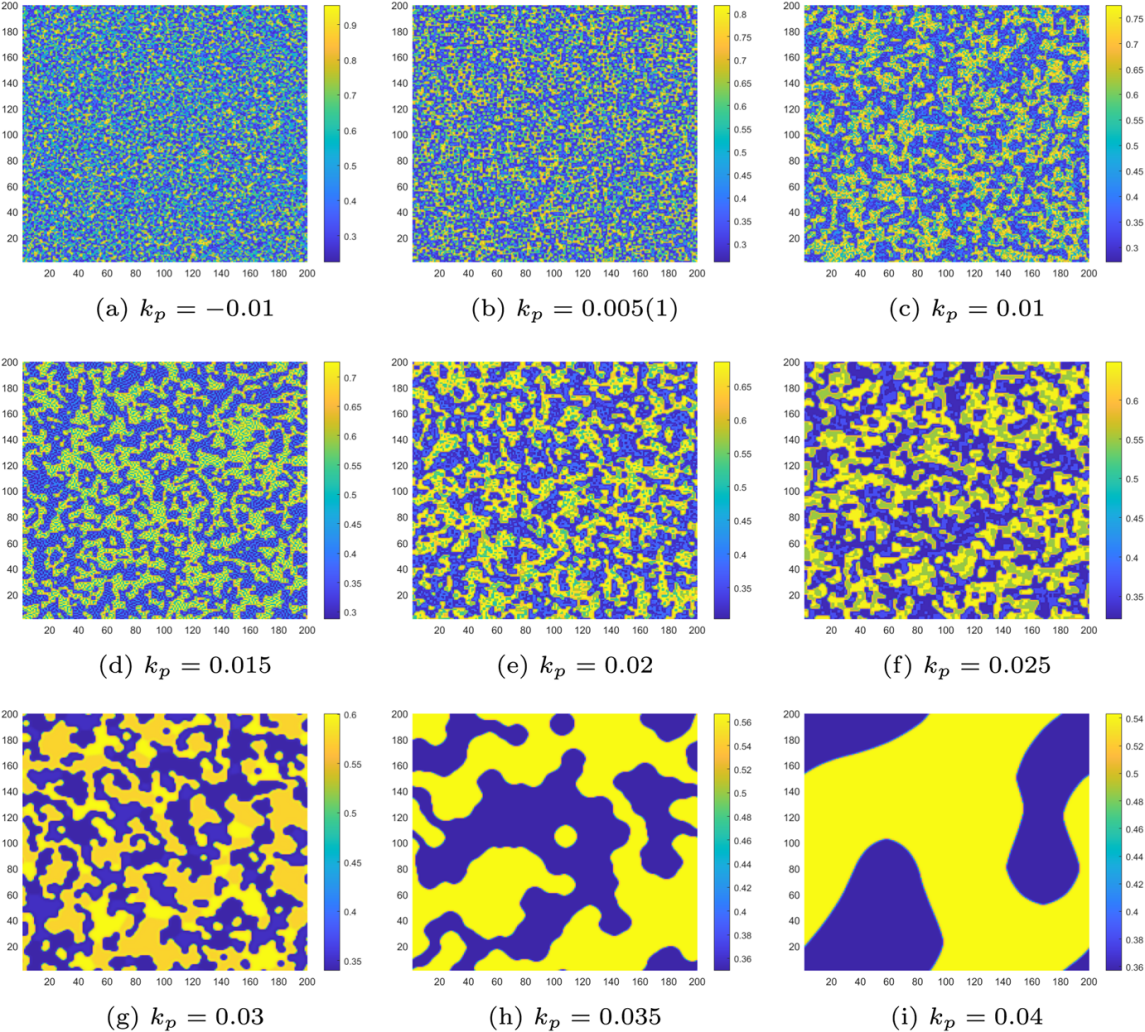
Turing patterns induced by flip-self-diffusion-Turing instability mechanism at $\tau =5.15$ change with control parameter $k_p$ when $k_d=0$

Firstly, we consider the case of $k_d=0, k_p \ne 0$. From Fig. 14, we can observe that as the control parameters $k_p$ increase, the pattern gradually transitions from the complex and fragmented state in Fig. 14a to a mode with only two state switches in Fig. 14h. When the control parameter $0.035\le k_p<0.055$, the pattern remains in the mode of two state switches until $k_p$ increases to 0.055. At this point, the Turing pattern becomes uniform, indicating that the distribution of algae *a* is uniform. In this case, we can also consider $k_p=0.055$ as the minimum control required to maintain the uniform distribution of algae *a*.

**Fig. 15:**
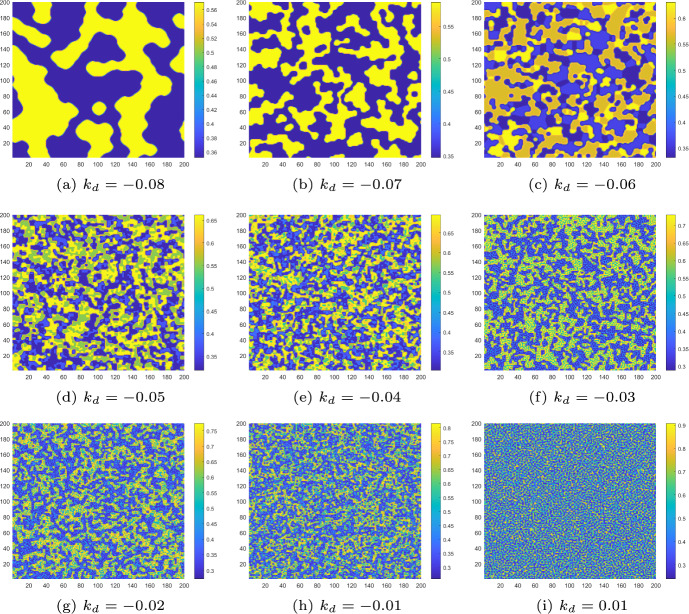
Turing patterns induced by flip-self-diffusion-Turing instability mechanism at $\tau =5.15$ change with control parameter $k_d$ when $k_p=0$

Secondly, we consider the case of $k_p=0, k_d \ne 0$. From Fig. 15, we can observe that as $k_d$ gradually increases, the pattern becomes increasingly fragmented, indicating that the distribution of algae *a* becomes more uneven. This is detrimental to the growth of mussel and, consequently, to the stability of the ecosystem in the aquaculture area. When $k_d$ is between $-0.14$ and $-0.07$, the Turing patterns maintain a mode of two state switches. Until $k_d$ decreases to $-0.14$, the pattern becomes uniform, identical to Fig. 7a. In this case, we can consider $k_d=-0.14$ as the maximum control that maintains the uniform distribution of algae *a*.

**Fig. 16:**
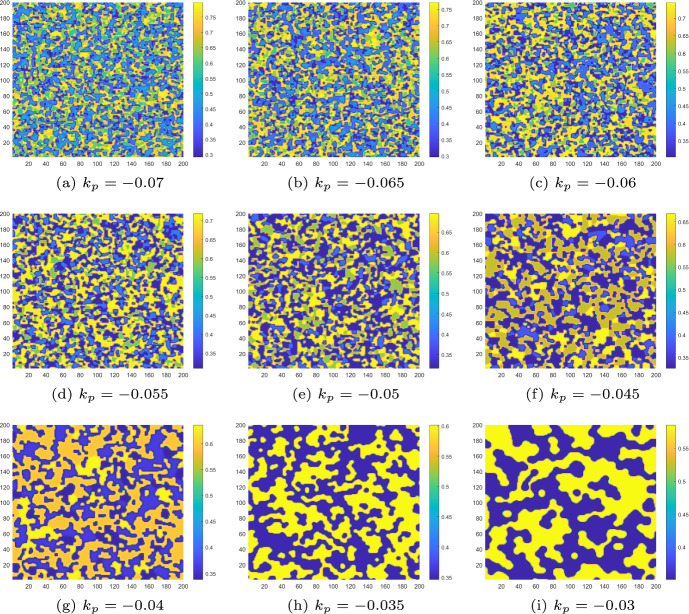
Turing patterns induced by flip-self-diffusion-Turing instability mechanism at $\tau =5.15$ change with control parameter $k_p$ when $k_d=-0.1$

Thirdly, we consider the case of $k_d=-0.1, k_p \ne 0$. From Fig. 16, it can be seen that as the control parameter $k_p$ increases, the fragmented state of the Turing pattern gradually diminishes. When $k_p=-0.001$, the pattern becomes completely uniform, like what is shown in Fig. 7a, indicating that the distribution of algae *a* is very uniform at this point. Furthermore, compared to the first case where $k_p=0.055$, the control parameter $k_p$ only needs to be applied to 0.001 in order to maintain the uniform distribution of algae *a*. This indicates that simultaneously adjusting both control parameters $k_p$ and $k_d$ can more effectively maintain the uniform distribution of algae *a*.

**Fig. 17:**
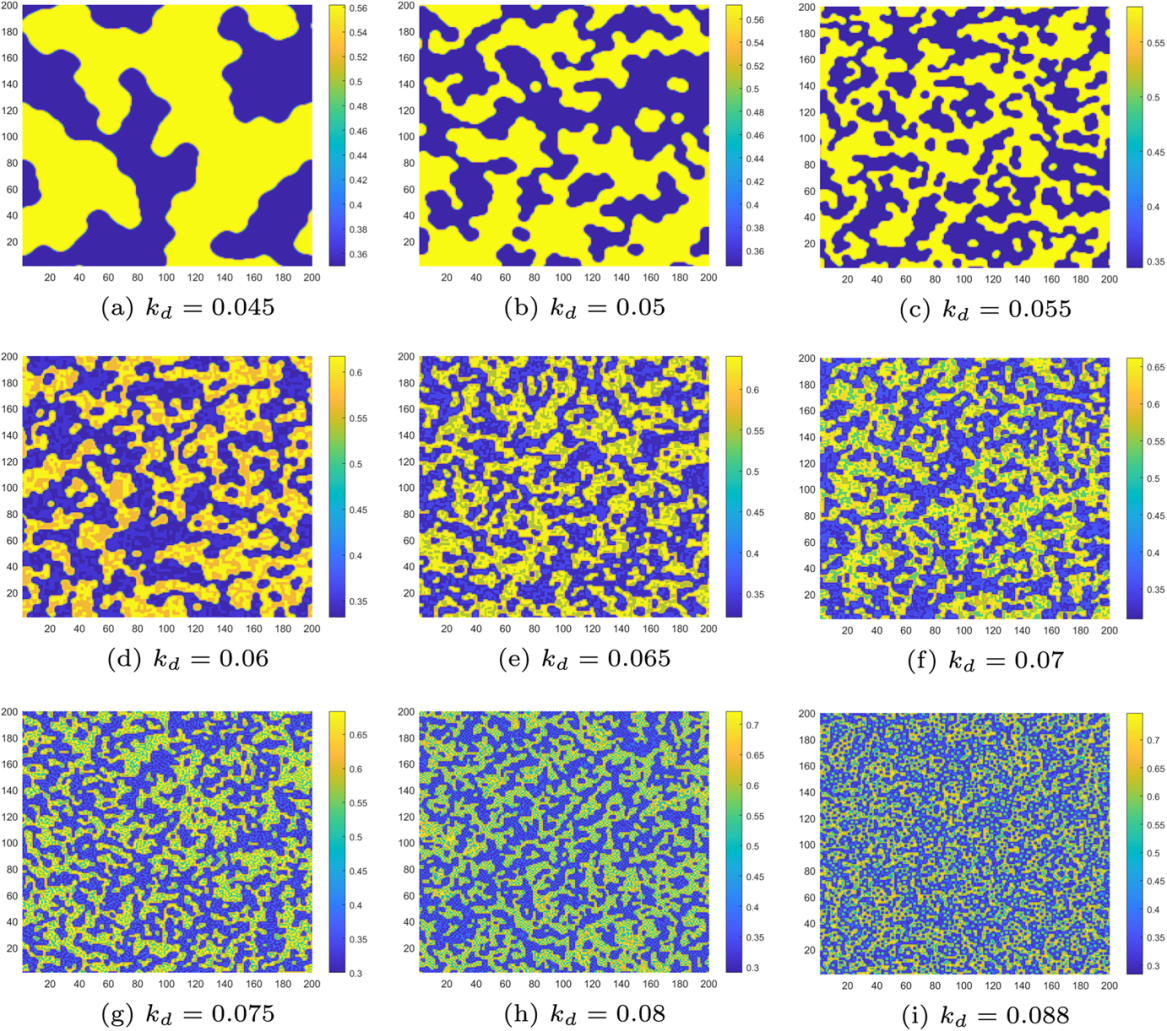
Turing patterns induced by flip-self-diffusion-Turing instability mechanism at $\tau =5.15$ change with control parameter $k_d$ when $k_p=0.045$

Finally, we consider the case of $k_p=0.045$, $k_d \ne 0$. From Fig. 17, it can be observed that the smaller $k_d$ is, the more favorable it is for the uniformity of the Turing patterns, which in turn helps maintain the uniform distribution of algae *a*. When $k_d=0.005$, the pattern becomes completely uniform, like as shown in Fig. 7a. Similarly, compared to the second case of $k_d=-0.14$, a control intensity of only 0.005 for $k_d$ is sufficient to ensure the uniform distribution of algae *a*.

In fact, the last two cases indicate that for the Turing patterns induced by flip-self-diffusion-Turing instability mechanism at $\tau =5.15$, $k_p=-0.001$ and $k_d=-0.1$, and $k_p=0.045$ and $k_d=0.005$, are two most effective and cost-efficient regulation strategies.

## Conclusions

This paper estabilished a spatiotemporally discretized CMLs model for a M-A system incorporating both self-diffusion and cross-diffusion. Through linear stability analysis, bifurcation theory, and the center manifold theorem, we investigated the stability and classification of fixed points in the CMLs model, including saddle, stable node, and stable degenerate node. Meanwhile, the conditions for the occurrence of flip bifurcation and Turing bifurcation were also derived. Notably, to mitigate instability arising from multi-period oscillations and chaotic fluctuations in algae population density–while ensuring a uniform spatiotemporal distribution of algae without compromising mussel growth and reproduction, we introduce a PD control into the CMLs model for the first time to regulate the system’s dynamical behaviors.

Through numerical simulations, we explored the rich temporal dynamics of the CMLs model, including period-doubling and chaos. The occurrence of these two dynamical regimes suggests that the population density of algae *a* undergoes unstable oscillations and enters a chaotic state, and by applying a PD control, the occurrence of the flip bifurcation can be effectively delayed. By plotting the maximum Lyapunov exponent, it is clearly demonstrated that this control effectively postpones the onset of chaos, thereby ensuring longer stability of algae population density.

At the same time, spatiotemporal patterns induced by five distinct mechanisms which we list for completeness: pure self-diffusion-Turing instability, pure cross-diffusion-Turing instability, flip-self-diffusion-Turing instability, flip-cross-diffusion-Turing instability, and chaos-self-diffusion-Turing instability were presented. With respect to patterns induced by flip-self-diffusion-Turing instability and flip-cross-diffusion-Turing instability mechanisms, we observed that, as the monitoring time $\tau$ increases, the pattern evolves from an initially uniform state to a two-state switching behavior, then to a four-state switching, and continues in this manner, becoming increasingly fragmented. This also implies that the distribution of algae *a* evolves and turns increasingly uneven, which is unfavorable for mussel’s growth. Notably, chaos-self-diffusion-Turing instability mechanism gives rise to disordered spatial patterns, indicating a loss of regular structure. Pure self-diffusion-Turing instability and pure cross-diffusion-Turing instability mechanisms instead induce patterns that exhibits a mosaic-like structure that is denser. However, regardless of the pattern induced by either Turing instability mechanism, the application of PD control is able to impose uniformity. That is, no matter how complex the spatiotemporal distribution of algae is, control can make it regularly distributed in space, reduce the Turing instability region, thus ensuring the stable growth of mussels and contributing to overall ecosystem stability.

We also uncovered variation trends between the flip bifurcation threshold and the PD control parameters. When $k_d$ is fixed, the flip bifurcation threshold value $\tau _{f}$ increases with the increase of $k_p$. While $k_p$ is fixed, the flip bifurcation threshold value $\tau _f$ decreases with the increase of $k_d$. Furthermore, by observing the $Z_m$–$\tau$ diagram with different values of $k_p$ and $k_d$, the Turing bifurcation threshold $\tau '$ increases with the increase of $k_p$ when $k_d$ is fixed; and increases with the increase of $k_d$ when $k_p$ is fixed. Moreover, the evolution of Turing patterns with different control parameters is also provided in different cases. Specifically, when $k_d=0$, the patches in the pattern become larger as $k_p$ increases, indicating that a higher $k_p$ promotes a more uniform distribution of algae *a*; when $k_p=0$, as $k_d$ increases, the patches in the pattern become smaller, indicating that a higher $k_d$ leads to more complex patterns and a more uneven distribution of algae, which is unfavorable for mussel’s growth. It is worth noting that when $k_d=-0.1$ is fixed, the pattern becomes even at $k_p=-0.001$ (instead of 0.055); and when $k_p=0.45$, the pattern becomes even at $k_d=0.005$ (instead of control strength like 0.14), which indicates that dual-parameter regulation is more effective at promoting a uniform distribution of algae, thereby facilitating mussel’s growth.

Finally, let us emphasise that although the proposed PD control strategy has been shown to effectively enhance the stability of the system and suppress undesired fluctuations, it is important to recognize the potential costs associated with its implementation. In ecological contexts, such control measures may require additional energy input, human or management effort, and other ecological interventions, which could limit their practical feasibility. Therefore, any application of these strategies should carefully consider the trade-offs between the achieved stability and the associated resource expenditure. Moreover, the long-term ecological impacts of sustained interventions should also be taken into account, as excessive control efforts could potentially alter ecosystem dynamics. Future research could further investigate optimization approaches that maintain the desired level of stability while minimizing energy consumption and management efforts, as well as explore adaptive control schemes that respond to changing environmental conditions in a cost-effective manner. This is essential for sustainability and practicality.
